# Extracellular ATP Hydrolysis Inhibits Synaptic Transmission by Increasing pH Buffering in the Synaptic Cleft

**DOI:** 10.1371/journal.pbio.1001864

**Published:** 2014-05-20

**Authors:** Rozan Vroman, Lauw J. Klaassen, Marcus H.C. Howlett, Valentina Cenedese, Jan Klooster, Trijntje Sjoerdsma, Maarten Kamermans

**Affiliations:** Netherlands Institute for Neuroscience, Amsterdam, the Netherlands; University of Washington, United States of America

## Abstract

Neuronal computations strongly depend on inhibitory interactions. One such example occurs at the first retinal synapse, where horizontal cells inhibit photoreceptors. This interaction generates the center/surround organization of bipolar cell receptive fields and is crucial for contrast enhancement. Despite its essential role in vision, the underlying synaptic mechanism has puzzled the neuroscience community for decades. Two competing hypotheses are currently considered: an ephaptic and a proton-mediated mechanism. Here we show that horizontal cells feed back to photoreceptors via an unexpected synthesis of the two. The first one is a very fast ephaptic mechanism that has no synaptic delay, making it one of the fastest inhibitory synapses known. The second one is a relatively slow (τ≈200 ms), highly intriguing mechanism. It depends on ATP release via Pannexin 1 channels located on horizontal cell dendrites invaginating the cone synaptic terminal. The ecto-ATPase NTPDase1 hydrolyses extracellular ATP to AMP, phosphate groups, and protons. The phosphate groups and protons form a pH buffer with a pKa of 7.2, which keeps the pH in the synaptic cleft relatively acidic. This inhibits the cone Ca^2+^ channels and consequently reduces the glutamate release by the cones. When horizontal cells hyperpolarize, the pannexin 1 channels decrease their conductance, the ATP release decreases, and the formation of the pH buffer reduces. The resulting alkalization in the synaptic cleft consequently increases cone glutamate release. Surprisingly, the hydrolysis of ATP instead of ATP itself mediates the synaptic modulation. Our results not only solve longstanding issues regarding horizontal cell to photoreceptor feedback, they also demonstrate a new form of synaptic modulation. Because pannexin 1 channels and ecto-ATPases are strongly expressed in the nervous system and pannexin 1 function is implicated in synaptic plasticity, we anticipate that this novel form of synaptic modulation may be a widespread phenomenon.

## Abstract 2

A slow mechanism of retinal synaptic inhibition involves hydrolysis of ATP released from pannexin 1 channels (from the tips of horizontal cell dendrites); the resulting protons and phosphates acidify the synaptic cleft, which inhibits neurotransmitter release.

## Author Summary

At the first retinal synapse, specific cells—horizontal cells (HCs)—inhibit photoreceptors and help to organize the receptive fields of another retinal cell type, bipolar cells. This synaptic interaction is crucial for visual contrast enhancement. Here we show that horizontal cells feed back to photoreceptors via a very fast ephaptic mechanism and a relatively slow mechanism. The slow mechanism requires ATP release via Pannexin 1 (Panx1) channels that are located on HC dendrites near the site where photoreceptors release the neurotransmitter glutamate to HCs and bipolar cells. The released ATP is hydrolyzed to produce AMP, phosphate groups, and protons; these phosphates and protons form a pH buffer, which acidifies the synaptic cleft. This slow acidification inhibits presynaptic calcium channels and consequently reduces the neurotransmitter release of photoreceptors. This demonstrates a new way in which ATP release can be involved in synaptic modulation. Surprisingly, the action of ATP is not purinergic but is mediated via changes in the pH buffer capacity in the synaptic cleft. Given the broad expression of Panx1 channels in the nervous system and the suggestion that Panx1 function underlies stabilization of synaptic plasticity and is needed for learning, we anticipate that this mechanism will be more widespread than just occurring at the first retinal synapse.

## Introduction

Natural scenes contain a large amount of redundant information both in space and time. For optimal coding of the visual scene, most of these redundancies need to be removed. The inhibitory interaction between horizontal cells (HCs) and cones is the first step in this process. HCs estimate the global properties of the stimulus. This information is subtracted from the local information sampled by the cones. The result is a strong reduction of redundant information in the cone output signal. In order to reduce spatial redundancies, this feedback mechanism needs to be very fast. If this were not the case, the surround of the bipolar cell receptive field would lag the center for moving stimuli. In contrast, to reduce temporal redundancies, the inhibition needs to be very slow; otherwise, long-lasting activity would not be removed selectively. In this article we show that these two seemingly incompatible requirements are merged into a feedback mechanism that consists of a very fast ephaptic component and a very slow component that modulates the synaptic efficiency by changing the pH buffering in the synaptic cleft of cones.

Cones project to HCs that are strongly coupled to each other electrically and provide negative feedback to the cones. Hyperpolarization of HCs by light shifts the activation potential of the presynaptic Ca^2+^ current (I_Ca_) in cones to more negative potentials [1]–[5], leading to a larger I_Ca_. This increases the Ca^2+^ concentration in the cone synaptic terminal so more glutamate is released. This modulation is not mediated by GABA [1],[3]. Two hypotheses have been put forward to account for the modulation of I_Ca_: an ephaptic mechanism [6]–[8] and a proton-mediated mechanism [4],[9],[10].

The ephaptic feedback mechanism depends on connexin (Cx) hemichannels and possibly pannexin 1 (Panx1) channels expressed at the tips of HC dendrites [6],[7],[11],[12]. These channels form large pores in the cell membrane, leading to an inward current. Given the finite resistance of the synaptic cleft, this inward current creates a slight negativity in the synaptic cleft. This negativity is sensed by the voltage-gated Ca channels of the cones as a slight depolarization of the cone membrane potential. HC hyperpolarization will increase the inward current through the Cx hemichannels, thus increasing the negativity in the synaptic cleft. This will be visible as an inward current in voltage clamped cones, reflecting a shift of I_Ca_ to more negative potentials. A major unresolved issue with the ephaptic feedback hypothesis is that it predicts a very fast feedback signal, whereas other studies indicate a relatively slow process [13],[14].

The proton-mediated feedback hypothesis is based on the pH sensitivity of I_Ca_ [15]. The activation potential of I_Ca_ shifts to more positive potentials in an acidic condition and to more negative potentials in an alkaline condition. To test the proton-mediated feedback hypothesis, Hirasawa and Kaneko [4] added 10 mM HEPES to the medium to reduce pH changes in the synaptic cleft and were able to show that the feedback-induced shift of I_Ca_ was reduced and sometimes even absent. They suggested that hyperpolarized HCs take up protons, which leads to an alkalization of the synaptic cleft. However, these experiments were not conclusive as more recent research found that 20 mM HEPES also induced a number of nonspecific effects, such as intracellular acidification and a direct inhibition of Cx hemichannels [16]. Moreover, the mechanism responsible for the proposed changes in proton concentration in the synaptic cleft has not yet been identified.

The aim of this article is to determine the synaptic mechanisms underlying lateral inhibition in the vertebrate outer retina. We show that the negative feedback pathway from HCs to cones is a synthesis of an ephaptic feedback mechanism and a proton-mediated mechanism. The ephaptic mechanism is one of the fastest inhibitory systems known and is especially suitable for spatial redundancy reduction in a dynamic scene. The proton-mediated mechanism depends on extracellular hydrolysis of ATP. HCs release ATP via Panx1 channels located on their dendritic tips that invaginate the synaptic terminals of cones. Ecto-ATPases hydrolyze ATP, which generates protons and a phosphate pH buffer, leading to an acidification of the synaptic cleft that inhibits I_Ca_. This pathway is very slow (time constant of about 200 ms) and does not involve purinergic or adenosine receptors. It is especially suitable for reducing temporal redundancies. Our findings not only resolve the longstanding controversy about the mechanism of negative feedback from HCs to cones, they also demonstrate a novel mechanism of synaptic modulation involving ATP released from Panx1 channels.

## Results

**Figure 1:**
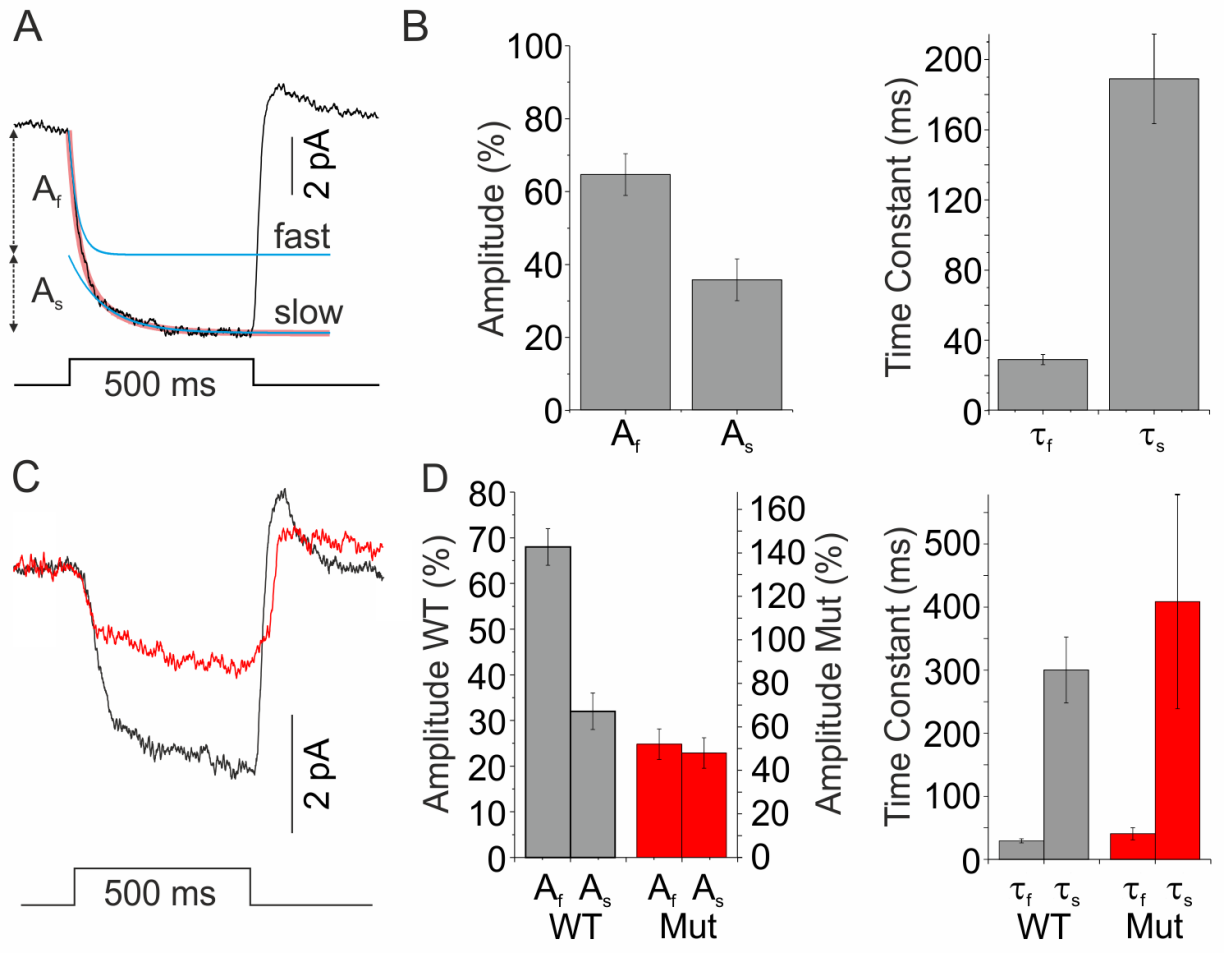
Feedback consists of a fast and a slow component. (A) Feedback response measured in a cone in goldfish retina. Cones were clamped at E_Cl_ (−50 mV) and saturated with a small spot of light. HCs were then hyperpolarized by a 500 ms full-field white light stimulus (4,500 µm). This induced a feedback inward current. The mean amplitude was 11.0±1.1 pA (*n* = 23). The onset of the feedback response could be fitted best by the sum of two exponentials (red line, sum of the exponential fits; blue lines, individual exponential fits). (B) The amplitude and time constants of the two exponential functions. The fast feedback component (τ_f_ = 29±3 ms; *n* = 23) had an amplitude (A_f_) that constituted 64±6% of the total feedback response. The remaining 36±6% of the feedback response (A_s_) was mediated by a process with a time constant (τ_s_) of 189±25 ms (*n* = 19). (C) Feedback responses in cones from wild-type (WT, black) and Cx55.5^−/−^ mutant zebrafish (red) were also best fitted by a double exponential function. (D) Compared to goldfish, WT zebrafish had a similar fast component to slow component amplitude ratio (gray: A_f_, 68±4%; A_s_, 32±4%; *n* = 13), whereas it shifted in Cx55.5^−/−^ mutants in favor of the slow feedback component (red: A_f_, 52±7%; A_s_, 48±7%; *n* = 9). The amplitude of the slow component of feedback in Cx55.5^−/−^mutants (1.62±0.39 pA; *n* = 9) did not significantly differ from WT (1.87±0.39 pA; *n* = 13; *p* = 0.67), showing that the slow component is independent of Cx55.5 hemichannels. Note that the amplitude axes for the WT and mutants are scaled such that it reflects the total reduction of feedback in the mutant relative to WT. The time constant of the fast component (τ_f_) did not differ significantly between WT and Cx55.5^−/−^ mutants (WT, 29.3±3.3 ms; *n* = 13; Cx55.5^−/−^, 40.6±9.6 ms; *n* = 9; *p* = 0.21) and was similar to goldfish. The time constant of the slow component (τ_s_) in WT and Cx55.5^−/−^ mutants did not differ significantly (WT, 300.2±52.3 ms; *n* = 13; Cx55.5^−/−^, 408.5±169.7; *n* = 9; *p* = 0.49) and was larger than in goldfish.

Negative feedback from HCs to cones was measured by voltage clamping cones in the isolated goldfish retina. The direct light response of the cone was saturated by a bright small spot and the retina was stimulated for 500 ms with a full field light flash to hyperpolarize HCs. Hyperpolarization of HCs leads to a shift of the activation potential of I_Ca_ of the cones to more negative potentials, which is visible as an inward current (Figures 1A and S1). On average this current was 11.0±1.1 pA (*n* = 23). Such feedback responses are not mediated by GABA [1],[3], but they can be modulated by a GABA_A_ pathway [17],[18]. In addition, as the small inward current induced by feedback represents the influx of Ca^2+^, a large Ca-dependent Cl current (I_Cl(Ca)_) is elicited by the cone feedback response [17],[19]. To exclude the interference of these confounds, we performed all our feedback experiments in the presence of 100 µM picrotoxin, a GABA_A_ receptor blocker, and clamped the cones at E_Cl_. Figure S1 shows that pure feedback responses can be recorded under these conditions.

The sum of two exponential functions fitted the light-induced feedback response significantly better than a single exponential function in 19 out of 23 cells (*p*<0.001) (Figure 1A; blue lines, the individual exponential functions; red line, sum of the two exponential functions). One exponential, the fast feedback component, rapidly decayed with a time constant of 29±3 ms (*n* = 23) and accounted for 64±6% of the feedback response. The second exponential, the slow feedback component, had a much slower time constant of 189±25 ms (*n* = 19) and accounted for the remaining 36±6% (Figure 1B). As shown in the supplemental material (Figure S2), the fast component of feedback was as fast as the HC responses, indicating HCs fed back to cones via a synapse that did not impose additional temporal filtering and had no synaptic delay (0.073±0.842 ms), making this synapse one of the fastest inhibitory processes in the nervous system. These features are consistent with an ephaptic feedback mechanism mediated by Cx hemichannels [6],[7],[16].

### The Fast and Slow Components of Feedback Are Mediated by Different Mechanisms

In zebrafish, the Cx hemichannels at the tips of the HC dendrites are formed by Cx55.5. Zebrafish lacking this Cx (Cx55.5^−/−^ mutant) have reduced feedback from HCs to cones [7]. Figure 1C shows feedback responses measured in cones of wild-type (WT, black) and Cx55.5^−/−^ mutant (red) zebrafish. The feedback-induced current was reduced by 48±11% in the Cx55.5^−/−^ mutant zebrafish (*n* = 22) compared to WT (*n* = 25). To quantify the contribution of both feedback components, double exponential functions were fitted through the feedback responses. Although the time constants of both components were independent of the genotype (Figure 1D), the amplitude of the fast feedback component was reduced in Cx55.5^−/−^ mutants such that the fast and slow feedback components now equally contributed to the feedback response. The amplitude of the slow feedback component in Cx55.5^−/−^ mutants (1.62±0.39 pA; *n* = 9) did not differ significantly from WT (1.87±0.39 pA; *n* = 13; *p* = 0.67), illustrating that the reduction of feedback in the Cx55.5^−/−^ mutant can be fully accounted for by the reduction of the fast feedback component. These experiments show that the fast component depends on Cx55.5, whereas the slow component does not.

**Figure 2:**
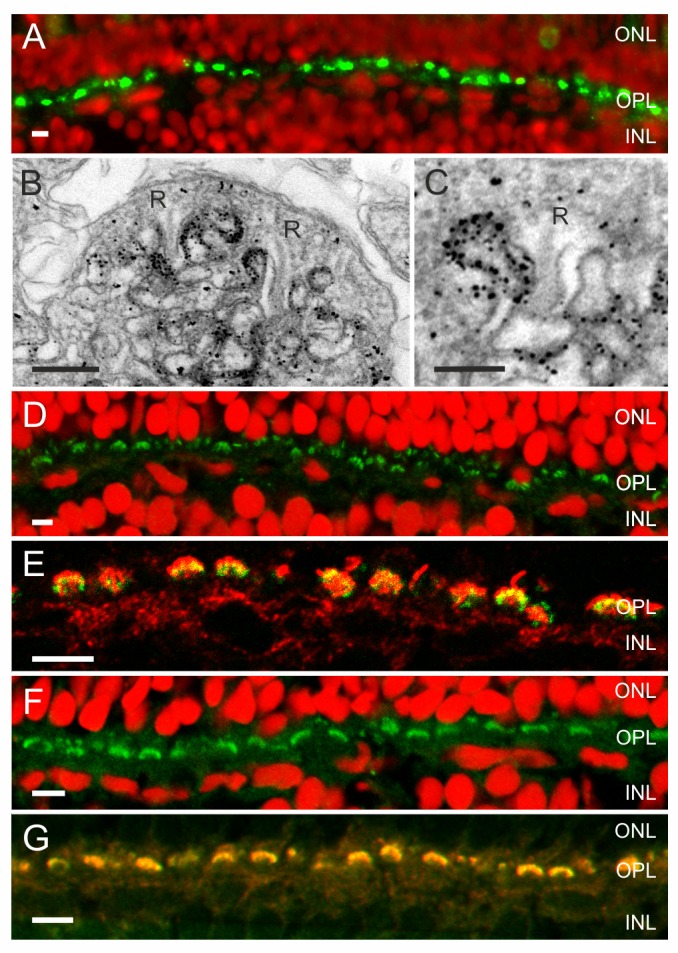
An ATP release mechanism and the enzymes needed to hydrolyze ATP and degrade adenosine are present in the synaptic complex of cones. (A) Fluorescent images of zebrafish retina showing Panx1-IR (green) and a nuclear stain (red) (from Prochnow et al. [11]). Panx1-IR is present in characteristic horseshoe-shaped structures indicative for processes invaginating the cone synaptic terminal. (B) Electronmicrograph of a cone synaptic terminal in zebrafish retina. Immunolabeling is restricted to HC dendrites flanking the synaptic ribbons (R). (C) Higher magnification of a HC dendrite lateral of the synaptic ribbon (R). Note that one HC dendrite is devoid of Panx1 labeling. (D) NTPDase1-IR (green) and a nuclear stain (red). NTPDase1-IR is also present in horseshoe-shaped structures characteristic for localization in HC dendrites. (E) Double labeling of NTPDase1 antibody with an antibody against GluR2 (green), a marker for HC dendrites [63],[64], indicated that NTPDase-1 (red) was specifically expressed on HC dendrites. (F) ADA-IR (green) was present in horseshoe-shaped structures. (G) ADA-IR colocalized with the GluR2-IR (green), indicating that ADA (red) is expressed on HC dendrites. Expression of ecto-ATPases in the photoreceptor synaptic terminal has been observed previously in rat [39], mouse, and zebrafish [61]. Scale bars in panels A, D, E, F, and G indicate 5 µm. Scale bar in panels B and C indicate 500 nm and 250 nm, respectively.

Previously we have shown that carbenoxolone, a Cx antagonist, blocks light-induced feedback responses in cones completely [6]. Interestingly, carbenoxolone is also a potent blocker of Panx1 channels [11]. Could Panx1 channels mediate the slow component of feedback? Panx1 channels are open at the resting membrane potential of HCs (−35 mV), reduce their conductance with hyperpolarization [11],[20],[21], and are modulated by intracellular Ca^2+^ [11],[22]. Figure 2A shows that Panx1-IR (green) was present as punctated labeling in the outer plexiform layer (OPL), suggesting Panx1 channels were localized at the tips of HC dendrites, which invaginate the cone's synaptic terminals [11]. Immuno-electron microscopy confirms this location. Panx1 labeling (black dots) was found in lateral elements flanking the synaptic ribbons (R) (Figure 2B and C).

**Figure 3:**
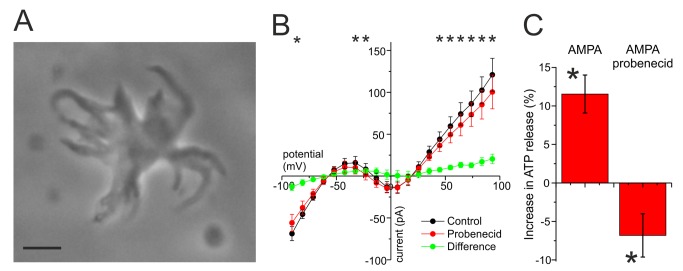
HCs release ATP. (A) Dissociated goldfish HC. This preparation consisted of about 90% of HCs. The remaining material was mostly debris. Scale bar, 20 µm. (B) The mean ± sem whole-cell IV relations of six dissociated HCs in conditions where potassium currents were inhibited by Cs. Application of 500 µM probenecid (red trace) reduces the conductance of the HCs. The green trace is the IV relation of the probenecid blocked current. This current has similar characteristics as the Panx1 current described by Prochnow et al. [11]. (C) Change in ATP release as measured with the luminescence assay. Depolarization of dissociated HCs by AMPA led to an increase in ATP release, whereas inhibition with probenecid decreased the release significantly. In probenecid, the ATP release was significantly lower than baseline, indicating that HCs in control conditions release ATP.

Next we determined whether Panx1 channels were active in HCs. Goldfish HCs were dissociated according to a modified protocol of Dowling et al. [23],[24] (Figure 3A). Such a preparation consists of about 90% of HCs, and it has been used previously to quantify GABA release by HCs [24]. Probenecid is a specific inhibitor of Panx1 channels that does not block Cx hemichannels [25] (see also Figure S3A). Whole-cell voltage clamp experiments on these dissociated HCs show that 500 µM probenecid blocks a current in the physiological membrane potential range with similar properties as previously described for Panx1 channels [11],[20],[21] (Figure 3B; black, control; red, probenecid; green, probenecid blocked current). On average probenecid reduced the whole-cell current statistically significant amounts at positive and negative potentials and in the physiological range (*n* = 6; asterisk means *p*<0.05). In three of the six cells, where stable recordings could be maintained, partial recovery was obtained. Similar results were obtained by application of another specific Panx1 blocker, 20 µM BB FCF (Figure S3B) [26]. The Panx1-mediated current was small, most likely because most of the Panx1 channels will have been lost during the dissociation process, as Panx1 is preferentially expressed at the tips of the HC dendrites.

Panx1 channels have been shown to mediate ATP release in many cell types [27],[28]. Can HCs release ATP upon depolarization? A luciferin–luciferase ATP detection assay was used to measure changes in the ATP release from the dissociated HCs. Bioluminescence was measured using a luminometer. After obtaining a baseline value, HCs were depolarized with 50 µM AMPA and the ATP concentration in the medium increased by 11.5±2.5%. The subsequent addition of 100 µM probenecid decreased the ATP concentration to 6.8±2.8% below the baseline value (*n* = 74; *p*<0.001). These data show that upon depolarization HCs release ATP via Panx1 channels (Figure 3C).

Finally, we determined whether Panx1 channels are active under physiological conditions. Panx1 channels mediate a current with a reversal potential more positive than the dark resting membrane potential of HCs [28], suggesting that these channels keep the HCs slightly depolarized and that their closure should cause HCs to hyperpolarize. We found that this was indeed the case. Inhibiting Panx1 channels with 500 µM probenecid hyperpolarized HCs on average −8.2±1.8 mV (*n* = 9). HCs are part of a closed feedback loop with cones. Hyperpolarization of HCs will lead to an increase in feedback, leading to more glutamate release by the cones, which limits the extent to which HCs will hyperpolarize. To isolate the effect of Panx1 on the HC membrane potential, we opened this closed loop by applying 28 mM HEPES, which is known to inhibit feedback substantially [4],[10],[16]. When feedback was inhibited in this way, HCs hyperpolarized significantly more when 500 µM probenecid was applied, compared to its application while feedback was intact (−19.2±2.7 mV; *n* = 9; *p* = 0.0047). These experiments confirm that HCs express Panx1 channels that are functional at physiological membrane potentials.

### Could ATP Release by HCs Mediate the Slow Component of Feedback?

It has been suggested that HCs take up protons upon hyperpolarization and that the resulting increase in pH modulates the I_Ca_ of cones [4]. Extensive evidence indicates that feedback is inhibited by HEPES [4],[10],[16]. Could ATP be involved in modulating the pH in the synaptic cleft? ATP can be hydrolyzed to ADP and AMP by ecto-ATPases. These reactions generate protons and phosphate groups, which constitute a phosphate pH buffer with a pKa of 7.2. Therefore, ATP released by HCs might lead to an acidification of the synaptic cleft relative to the extrasynaptic medium (7.6–7.8) and an increase in the pH buffer capacity of the synaptic cleft. This leads to an inhibition of I_Ca_ of the cones. Upon hyperpolarization of HCs, Panx1 channels will reduce their conductance and the release of ATP will decrease, resulting in a drop of the pH buffer capacity and an alkalization of the synaptic cleft. The inhibition of I_Ca_ will be relieved. This hypothesis depends on three critical aspects: (1) release of ATP, (2) hydrolysis of ATP, and (3) changes in pH and pH buffer capacity in the synaptic cleft. The dependence of the slow component of feedback on these three aspects was tested next.

**Figure 4:**
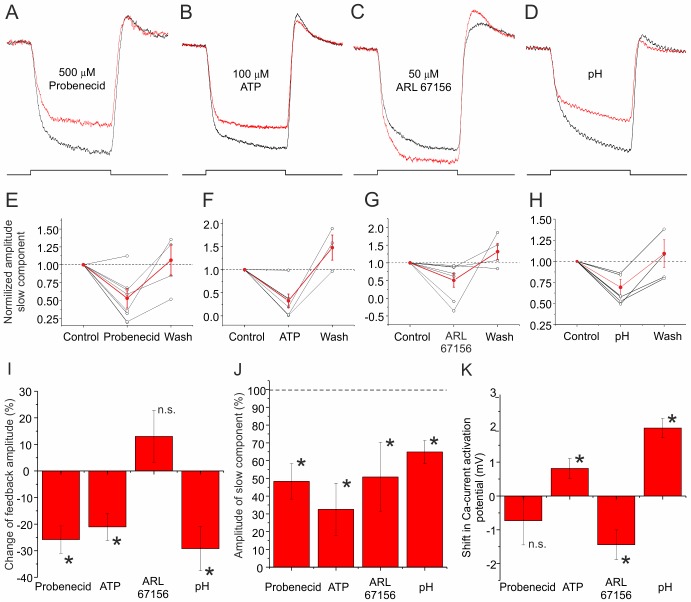
Pharmacological profile of the slow component of feedback. (A) Normalized mean feedback responses of nine cones in control (black) and in 500 µM probenecid (red). The slow component present in control conditions was reduced by probenecid. (B) Normalized mean feedback responses in nine cones in control (black) and in 100 µM ATP (red). ATP blocked the slow component of feedback, indicating the slow component depends on ATP. Note this concentration of ATP does not block Panx1 channels [20]. (C) Normalized mean feedback responses in seven cones in control (black) and in 50 µM ARL67156, a blocker of NTPDase (red). The slow component is reduced by application of ARL67156, indicating that it is mediated by ATP hydrolysis. Feedback response amplitudes were reduced by ATP application (B), but not by ARL67156 application (C), consistent with the notion that a pH buffer generated by ATP hydrolysis inhibits I_Ca_ of the cone by keeping the synaptic cleft slightly acidic. (D) Normalized mean feedback responses in six cones in control (pH 7.6; black) and when the superfusate pH was 7.2 (red). The slow component is strongly reduced when the pH gradient between the synaptic and the extrasynaptic compartments is removed. (E–H) The slow component amplitude for individual cells (black) used in (A–D) during control, drug application, and wash. The red line indicates the mean ± sem. In each case, the changes of the slow component amplitude were reversible. The figures also suggest that the slow feedback component may have increased slightly over time. (I) The mean ± sem change in the total feedback amplitude relative to control levels for the results shown in (A–D). Probenecid (*p* = 0.003), ATP (*p* = 0.0093), and pH (*p* = 0.0175) all reduced the feedback amplitude significantly. Only ARL67156 did not change the amplitude significantly (*p* = 0.2295). (J) The mean ± sem amplitude reduction for the slow component of feedback relative to control values for the results shown in (A–D). In each case, the amplitude reduction of the slow component was significant (probenecid, *p* = 0.0009; ATP, *p* = 0.036; ARL67156, *p* = 0.045; pH, *p* = 0.010). (K) The change in half activation potentials of I_Ca_ when 500 µM probenecid, 100 µM ATP, or 50 µM ARL67156 were present in the bath solution or when the extracellular pH was shifted to 7.2. Probenecid did not induce a significant shift of the activation potential of I_Ca_ (*n* = 9; *p* = 0.3375). ATP shifted the half activation potential to more positive potentials (*n* = 8; *p* = 0.0277), whereas ARL67156 shifted it to more negative potentials (*n* = 8; *p* = 0.0131). Lowering the extracellular pH led to a significant shift of I_Ca_ to positive potentials (*n* = 5; *p* = 0.0022).

Panx1 channels were inhibited with 500 µM probenecid, and feedback responses in cones were measured. Figure 4A shows mean responses (control, black; probenecid, red). Examples of the responses of individual cells are given in Figure S4A (wash, green). In the presence of probenecid, the amplitude of feedback responses reduced by 26±8% (*n* = 9; *p* = 0.0009) and the shape of the response became more square (Figure 4A and I). The slow component of feedback often reduced to such an extent that a two exponential fit could not be performed reliably. After application of probenecid, a single exponential function fitted the response significantly better than a double exponential function in seven of the nine cells tested, whereas this was the case for only one cell in the control condition. Therefore, to quantify the reduction of the slow component, we determined the difference in amplitude measured at 160 ms and 460 ms after stimulus onset (see Materials and Methods). Application of probenecid resulted in a 51±10% reduction of the slow component (*n* = 9; *p* = 0.003) (Figure 4A and J). Figures 4E and S4A show that these effects were reversible.

To test whether the slow component of feedback was dependent on ATP, we applied 100 µM ATP, a concentration that does not block Panx1 channels [20], and measured feedback responses. The amplitude of the feedback response reduced by 21±5% (*n* = 6; *p* = 0.009) (Figures 4B,I and S4B) and became squarer in shape. The size of the slow component reduced by 67±15% (*n* = 6; *p* = 0.036) (Figure 4B and J) and recovered after washing out the drug (Figures 4F and S4B). These experiments indicate that ATP is able to modulate the slow component of feedback.

ATP hydrolysis by ecto-ATPases should lead to acidification. First we tested whether ecto-nucleoside triphosphate diphosphohydrolase (NTPDase1) was present in the cone synaptic cleft. Figure 2D shows NTDPase1-IR (green) in horseshoe-shaped structures in the OPL, at a similar localization as the Panx1-IR shown in Figure 2A. Double labeling of anti-NTPDase1 with an antibody against GluR2, the glutamate receptor expressed at the HC dendrites, resulted in complete overlap (Figure 2E). This shows that NTPDase1 is expressed at the tips of HC dendrites. Because NTPDase1 is a protein with two membrane spanning domains with its catalytic domain located extracellularly, these experiments show that enzymes that hydrolyze extracellular ATP are present within the synaptic complex of cones, allowing for the synthesis of protons and a phosphate buffer and thus acidification of the synaptic cleft. ATP hydrolysis eventually leads to the formation of adenosine. Adenosine deaminase (ADA) is the enzyme that extracellularly degrades adenosine to inosine. Figure 2F shows ADA-IR in similar horseshoe-shaped structures in the OPL, as was found for NTDPase1-IR. Double labeling with an antibody against the glutamate receptor GluR2 shows strong co-localization (Figure 2G), suggesting that ADA is also expressed on the HC dendrites invaginating the cone synaptic terminal. These two enzymes allow for effective hydrolyzation of ATP in the synaptic cleft.

Is the hydrolysis of ATP an essential step in the feedback pathway? Blocking NTPDase1 with 50 µM ARL67156, a specific blocker of NTPDases [29], made the feedback response more square and reduced the amplitude of the slow component of feedback (49±20%; *n* = 7; *p* = 0.045) (Figures 4C,J and S4C). This effect was reversible (Figures 4G and S4C). Contrary to the effect seen when ATP was applied, the amplitude of the total feedback response did not decrease but may even have increased (113±10%; *n* = 7; *p* = 0.23) (Figure 4I). This experiment shows that the hydrolysis of ATP is involved in the generation of the slow component of feedback.

Finally, we tested whether the slow component of feedback depends on the pH gradient between the synaptic (pH 7.2) and the extrasynaptic (pH 7.6) compartments. We decreased the pH of the Ringer's solution by changing the ratio of CO_2_ and O_2_, with which the Ringer's solution was gassed. This might cause two changes: the pH gradient is reduced and the pH in the synaptic cleft might reduce. Figures 4D,J and S4D show that the slow component was reduced by acidifying the extrasynaptic compartment (35±6%; *n* = 6; *p* = 0.010). This effect was reversible (Figures 4H and S4D). The feedback amplitude was reduced by 29±8% (*n* = 6; *p* = 0.017) (Figure 4D and I).

The pharmacological manipulations we applied are expected to change the pH and the pH buffer capacity in the synaptic cleft. Barnes and Bui [15] showed that pH modulates the activation potential of I_Ca_ of cones. Therefore, it is expected that the half activation potential of I_Ca_ will have shifted in the various pharmacological conditions used in this study. According to the proposed hypothesis, ATP should acidify the synaptic cleft, whereas ARL67156 alkalizes it, predicting that I_Ca_ will shift to more positive potentials in ATP and to more negative potentials in the ARL67156. Indeed this was found. Application of ATP shifted the activation of I_Ca_ 0.8±0.3 mV (*n* = 8; *p* = 0.027) to more positive potentials, whereas ARL67156 shifted the activation of I_Ca_ −1.4±0.4 mV (*n* = 8; *p* = 0.013) to more negative potentials (Figure 4K). Consistent with these results is that acidifying the extracellular medium also leads to acidification of the synaptic cleft and thus to a shift of the activation potential of I_Ca_ to positive potentials (2.0±0.3 mV; *n* = 5; *p* = 0.002). Interestingly, application of probenecid did not lead to a significant shift of I_Ca_ (−0.7±0.7 mV; *n* = 9; *p* = 0.34).

It has been suggested the vacuolar H^+^-ATPase (V-ATPase) in the HC plasma membrane is involved in mediating negative feedback from HCS to cones [30]. However, no direct effects of blocking V-ATPase on the feedback-induced modulation of I_Ca_ in cones have been published. To see whether negative feedback indeed depends on the activity of V-ATPase, we applied the specific V-ATPase blocker bafilomycin A1 (BFA1). BFA1 did not significantly affect either the total feedback amplitude (113±11%; *n* = 8; *p* = 0.27) or the slow component (138±22%; *n* = 8; *p* = 0.14) of the feedback response, making it unlikely that V-ATPase is involved in feedback-induced modulation of the cone I_Ca_ within the physiological range.

### How Does the Panx1/ATP Feedback Mechanism Affect HCs?

**Figure 5:**
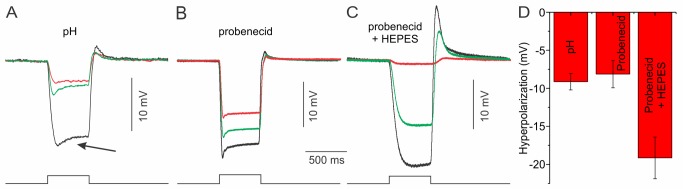
HC responses in various pharmacological conditions. (A) Acidification of the extrasynaptic medium reduces the rollback response in goldfish HCs (arrow) (*n* = 4) and hyperpolarizes the HC membrane potential (see D) (black, control; red, pH 7.2; green, wash). This indicates that the slow component of feedback contributes to the rollback response in HCs. (B) Application of probenecid also led to a slight reduction of the rollback response (*n* = 18) and hyperpolarization of the HC membrane potential (see D). The rollback response never disappeared completely (black, control; red, probenecid; green, wash). (C) Application of probenecid in a Ringer's solution containing 28 mM HEPES. In this condition, the HC light responses were almost completely blocked and HC hyperpolarized strongly [see D; black, HEPES; red, HEPES+probenecid; green, HEPES (wash)]. (D) Mean ± sem HC membrane hyperpolarization induced by the various pharmacological manipulations.

It is expected that negative feedback from HCs to cones will affect the kinetic properties of HCs. HC responses consist of a fast hyperpolarization followed by a slow rollback response (Figure 5A, left trace, arrow). It has been suggested that the HC rollback response correlates, at least partly, with negative feedback from HCs to cones [13],[31],[32]. However, some caution is warranted, as the rollback response is also influenced by many other processes, such as the transient nature of the cone response [33],[34] and voltage-gated currents [35],[36] in HCs. Because the rollback response is relatively slow, the slow component of feedback might be influenced most by the slow component. First, we tested whether the rollback response depended on the pH gradient in the synaptic cleft. Figure 5D shows that changing the pH of the extrasynaptic medium from 7.6 to 7.2 hyperpolarized HCs (−9.1±1.1 mV; *n* = 4). In all four cells tested, the rollback response reduced but did not disappear completely and recovered after returning to pH 7.6 (Figure 5A; black, control; red, pH 7.2; green, wash). This shows that the HC rollback response depends on the pH gradient in the synaptic cleft just as the slow component of feedback does (Figures 4D,H,J and S4E).

Next we tested if application of 500 µM probenecid would also reduce the rollback response. Application of probenecid hyperpolarized the HC membrane potential (Figure 5D), and even though there was a high degree of variability in the rollback responses, it was reduced in 10 out of 18 HCs (Figure 5B; black, control; red, probenecid; green, wash). However, the rollback response never completely disappeared. Note that this result resembles the effect of probenecid on the slow component of feedback, which only reduced by about 45% but never disappeared completely (Figure 4J). This is to be expected as probenecid is only a partial blocker of Panx1 channels [37]. Furthermore, because probenecid does not affect Cx hemichannels, feedback via Cx hemichannels is still present. This might account for the remaining rollback response in HCs. To test this, we determined the effect of probenecid in conditions when a major part of feedback was blocked by 28 mM HEPES. HEPES most likely has two effects on the feedback system. It inhibits the proton-mediated feedback by buffering the pH in the synaptic cleft, and it leads to the closure of Cx hemichannels [16]. When 28 mM HEPES was present in the medium, applying probenecid hyperpolarized HCs significantly more (Figure 5D) and no sign of rollback could be seen (Figure 5C; black, HEPES; red, HEPES+probenecid; green, HEPES). This result indicates that feedback is blocked (almost) completely in this condition. These experiments indicate that the rollback response depends, at least partly, on the slow feedback component.

### Purinergic Signaling Is Not Involved in Negative Feedback from HCs to Cones

**Figure 6:**
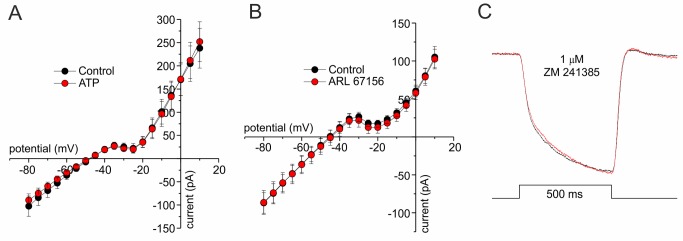
Purinergic receptor activation does not underlie feedback from HCs to cones. (A) A whole-cell IV relation for cones in control condition (black) and with 100 µM ATP (red; *n* = 5). ATP does not activate a nonspecific cation conductance. (B) A whole-cell IV relation for cones in control condition (black) and 50 µM ARL67156 (red; *n* = 5). ARL67156 did not lead to the activation of a nonspecific cation conductance. (C) Mean feedback responses in control (black) and in 1 µM ZM 241385 (red; *n* = 4), a blocker of the A2 receptor. The response amplitude and kinetics of the feedback response were not significantly affected, indicating that feedback is not mediated by A2 receptors.

In many systems ATP released from Panx1 channels activates purinergic receptors [38]. Some evidence exists for expression of P2X [39] and P2Y [40] receptors on photoreceptors. Activation of P2X receptors by ATP should induce a nonspecific cation current [41]. Whole-cell IV relations were constructed for cones in control conditions and when 100 µM ATP (*n* = 5) (Figure 6A) or 50 µM ARL67156 (*n* = 5) (Figure 6B) was added to the bath solution. Neither ATP nor ARL67156 appeared to modulate a cation conductance, making it unlikely that ATP acted directly on a P2X receptor. The data presented in Figure 4 also show that purinergic receptors are unlikely to be involved in mediating negative feedback from HCs to cones. Either applying ATP or blocking NTPDase1 with ARL67156 will increase the ATP concentration. If ATP affected feedback by modulating photoreceptor purinergic receptors, ATP and ARL67156 would have had similar effects. Although both ATP and ARL67156 reduced the slow component of feedback, the shift of I_Ca_ and the size of the total feedback response moved in opposite directions (Figure 4I, J, and K). This implies that ATP hydrolysis and not ATP itself exerts an effect on the slow component of feedback. In addition, A2 receptors have been suggested to modulate photoreceptor function [42]–[44]. We excluded a possible role for adenosine by measuring feedback in the presence of the specific A2 receptor blocker, ZM 241385, at a similar concentration as used by Stella and co-workers (Figure 6C) [42]–[44]. In this condition, neither the feedback amplitude nor its kinetics were affected (*p*>0.2; *n* = 4), indicating that A2 receptors do not mediate feedback.

## Discussion

This study demonstrates two extraordinary forms of synaptic inhibition. HCs feed back negatively to cones via two mechanisms: a very fast Cx-hemichannel-mediated ephaptic mechanism with no synaptic delay and a relatively slow mechanism that modulates the pH and pH buffer capacity in the synaptic cleft of the cones. This slow mechanism involves ATP release via Panx1 channels, ATP hydrolysis by ecto-ATPases, and is not purinergic. Inhibiting ATP release, adding ATP, and blocking the hydrolysis of ATP all inhibit the slow feedback component. Furthermore, changing the pH gradient between the synaptic compartment and the extracellular medium also inhibited the slow feedback component. These results are all consistent with a mechanism in which the pH and pH buffer capacity in the synaptic cleft of the cone is modulated by ATP released from HCs via Panx1 channels.

The mechanism of negative feedback has puzzled the retinal community for decades and various hypotheses have been put forward. None of these hypotheses could account for all experimental data. There were three major issues with the competing hypotheses: the kinetics of the feedback response were not accounted for, the pH-modulating mechanism was unknown, and the proposed proton mechanism was intrinsically unreliable. First, the kinetic features of the feedback responses have been resolved. Ephaptic feedback is extremely fast, and proton-mediated feedback is very slow. Second, as outlined below, the pH-modulating mechanism has been identified: Panx1 channels release ATP, which is hydrolyzed by NTPDase1 and leads to changes in pH and pH buffer capacity in the synaptic cleft. Finally, we will argue that changing the pH buffer capacity is a much more reliable form of proton-mediated signaling than changing the proton concentration in an unbuffered synaptic cleft.

### How Do the Fast and the Slow Feedback Mechanisms Interact?

**Figure 7:**
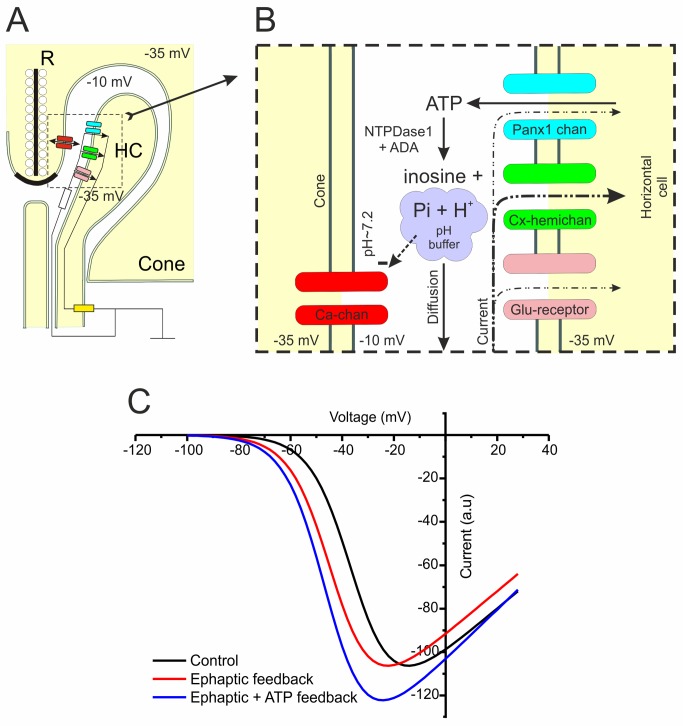
Schematic drawing of the proposed feedback mechanism. (A) The ephaptic mechanism. Cones continuously release glutamate in the dark via a ribbon (R) synapse optimized for sustained glutamate release. This release depends on the activity of presynaptic Ca^2+^ channels (red). HC dendrites end laterally to the cone synaptic ribbon. Cx hemichannels (green) and Panx1 channels (blue) are expressed on the dendrites of HCs. Because they are both nonspecific channels and are open at the physiological membrane potentials of HCs, a current will flow into the HCs. HC dendrites invaginate the cone synaptic terminal, which leads to a large extracellular resistance (white resistor). The current that flows into HCs via the Cx hemichannels and Panx1 channels has to pass this resistor, which will induce a slight negativity deep in the synaptic cleft. The result will be that the voltage-gated Ca^2+^ channels sense a slightly depolarized membrane potential. When HCs hyperpolarize, the current through the Cx hemichannels will increase and so will the negativity in the synaptic cleft leading to a further decrease of the potential sensed by the cone Ca^2+^ channels. This is the fast component of feedback. (B) The Panx1/ATP-mediated mechanism. Expanded view of the pre- and postsynaptic membranes of cones and HCs. ATP is released by HCs via Panx1 channels. Through a number of steps ATP is converted into inosine, protons, and a phosphate buffer with a pKa of 7.2. This makes the synaptic cleft acidic relative to the extrasynaptic medium (pH 7.6–7.8), which inhibits Ca^2+^ channels and shifts their activation potential to positive potentials. Closing the Panx1 channels prevents HCs from releasing ATP, thereby stopping the production of phosphate buffer, leading to an alkalization of the synaptic cleft. This alkalization disinhibits the Ca^2+^ channels and shifts their activation potential to negative potentials. This mechanism underlies the slow component of feedback. (C) Schematic representation of I_Ca_ of cones with and without feedback. The black trace shows the I_Ca_ of cones in the dark. The red trace shows I_Ca_ when only the ephaptic feedback is active, whereas the blue trace shows the I_Ca_ when both the ephaptic and Panx1/ATP-mediated feedback are active.

Figure 7 summarizes the proposed mechanism of negative feedback from HCs to cones. Glutamate receptors, Cx hemichannels, and Panx1 channels are expressed in the postsynaptic HC membrane. Voltage-gated Ca channels are expressed on the presynaptic cone membrane. In the dark, HCs and cones rest at about −35 mV. As I_Ca_ in cones is activated at that potential, cones release glutamate and HC glutamate receptors are activated, depolarizing HCs. Glutamate-gated channels, Cx hemichannels, and Panx1 channels are open in this condition and current flows into the HC. Because the extracellular space has a finite resistance, this current makes the potential deep in the synaptic cleft slightly negative. This is sensed by the voltage-gated Ca channels in the presynaptic membrane of the cones as a slight depolarization. The effect will be that the activation potential of I_Ca_ has shifted to more negative potentials relative to the cone's overall membrane potential [6],[7],[45].

At the same time, ATP is released via Panx1 channels. ATP is hydrolyzed to ADP, AMP, and eventually to adenosine by ecto-ATPases. This hydrolysis leads to an acidification of the synaptic cleft and the formation of a phosphate pH buffer (pKa of phosphate buffer is 7.2). Acidification of the synaptic cleft inhibits the cone I_Ca_ [15] and shifts its activation potential to more positive potentials. Interestingly, the ephaptic mechanism shifts the activation potential of I_Ca_ to more negative potentials, while the Panx1/ATP-mediated mechanism shifts it to more positive potentials. These two opposing mechanisms together set the activation potential of I_Ca_ in the dark (Figure 7C, black line). Hyperpolarization of HCs due to surround stimulation will have two effects on the system. First, the current flowing through the Cx hemichannels, the Panx1 channels, and the glutamate-gated channels will increase. This increases the negativity of the synaptic cleft, shifting I_Ca_ to more negative potentials (Figure 7C, red line). This process is as fast as the change in HC membrane potential and does not have a synaptic delay. In due time, the Panx1 channels will reduce their conductance and the release of ATP will diminish. Gradually the proton concentration and the pH buffer capacity in the synaptic cleft reduce, and the resulting alkalization disinhibits I_Ca_. As a consequence, I_Ca_ increases and its activation potential shifts to even more negative potentials (Figure 7C, blue line). This process is the slow feedback component and has a time constant of about 200 ms.

### The Fidelity of the Panx1/ATP Feedback Mechanism

The form of synaptic modulation we propose here is novel as it exerts its actions by regulating the local pH buffer capacity. Changing the pH buffer capacity consequentially changes the pH in the synaptic cleft. The feedback-induced pH change in the synaptic cleft is therefore indirect. This has an enormous advantage over direct modulation of the proton concentration in the synaptic cleft in an unbuffered system. To illustrate this, we calculated the number of protons involved in a feedback response. The maximal feedback-induced shift of I_Ca_ is about 9 mV [16], and a pH change of 0.1 units shifts I_Ca_ by 1 mV [15]. Here we show that about 35% of the total feedback current in cones is pH-mediated. This implies that the slow component of feedback is responsible for about 3 mV of the shift of I_Ca_, which translates to a pH change in the synaptic cleft of about 0.3 pH units. Vandenbranden [46] estimated that the volume of the extracellular space within the synaptic terminal of goldfish cones is 0.88 µm^3^. Given these numbers, a 0.3 pH unit change in the pH in the synaptic cleft would involve the movement of about 19 protons from the total pool of around 38 free protons. In an unbuffered system, this would produce an unreliable noisy signal. However, the phosphate buffer is most likely present in the range of hundreds of µM, making the number of free protons a well-controlled statistical parameter. On the other hand, if the synaptic pH was strongly buffered by static pH buffers, proton-mediated feedback would be largely inefficient. Any pH change induced by HC hyperpolarization would be counteracted by the activity of the static pH buffers. This suggests that changing the pH buffer capacity is possibly the only way proton-mediated synaptic transmission can work. In this way, a pH-signaling mechanism can be highly reliable and noise free.

In the dark, cones continuously release glutamate. Because glutamate and protons are co-released by the cones, this would lead to an acidification of the synaptic cleft in the dark and an alkalization in the light. Negative feedback leads to an increase in glutamate release and hence an increase in proton release as well—that is, an acidification. The opposite is found, suggesting that the pH near the Ca channels in the synaptic cleft depends more on HC activity than on cone activity. This is highly unexpected as glutamate is released continuously very close to the Ca channels of the cones. The reason why the pH in the synaptic cleft depends more on HC activity than on cone activity might be that the pH in the synaptic cleft is set by the pH buffer capacity, which is modulated by HCs, and not directly by proton release or uptake.

Noise reduction is of great importance for an optimally performing visual system. The feedback pathway from HCs to cones modulates the output of the photoreceptors. Any noise added at this level of the visual system will considerably decrease the visual performance of the whole visual system. In later processing stages, noise can be lowered by convergence and by distributing the signals over various parallel channels. Such options are not available for the photoreceptors. Both feedback mechanisms we describe here have low noise properties; for instance, neither of them depends on vesicular release of neurotransmitters or activation of postsynaptic receptors. This unusual feedback synapse thus seems to be optimally adapted for its function in the outer retina.

### Other Evidence for pH Changes in the Synaptic Cleft

Wang et al. [14] studied feedback-induced pH changes in the synaptic cleft of zebrafish cones using a fluorescent method. They found that HC depolarization leads to acidification of the synaptic cleft. The time course (τ∼200 ms) of this pH change is remarkably similar to the time course of the second feedback component (τ_s_ = 189±25 ms) we find. Also, the size of the pH change they describe is very similar to the pH change we predict. In other words, the results of Wang et al. [14] are fully consistent with our experimental data. Wang et al. [14] did not find the fast component of feedback we have described here. However, they solely used fluorescent measurements to study pH changes in the synaptic cleft. As such, these types of experiments would be unable to detect the fast feedback component we describe, as it is mediated by an ephaptic mechanism and not by a pH-dependent mechanism.

Wang et al. [14] suggested that the pH changes are mainly due to V-ATPase activity because they could inhibit the feedback-induced pH changes in the synaptic cleft with BFA1. We tested the effect of BFA1 on light-induced feedback responses measured in the cones and found that neither the fast nor slow feedback component was affected by BFA1. These results indicate that light-induced feedback does not depend on V-ATPase activity. This discrepancy may relate to the transgenic approach Wang et al. [14] used. They studied pH changes in the synaptic cleft in a transgenic zebrafish line that expressed Na channels (FaNaChannels) in HCs. To depolarize the HCs, FMRFamide, an agonist for the FaNaChannels, was applied. Because the reversal potential for sodium is very positive, it is highly likely that HCs in their experiments were depolarized to potentials far outside their physiological operating range. The HC membrane potentials might even have become positive during the depolarization, which is a very unphysiological condition. We show that under physiological conditions BFA1 does not significantly affect feedback, making it unlikely that V-ATPase has a role in negative feedback from HCs to cones.

### The Advantage of Two Feedback Pathways

The question arises as to why two feedback mechanisms are present in the outer retina instead of one. To reduce spatial redundancies in the visual scene, the mean activity of all cones within the large receptive field of HCs is subtracted from the output of individual cones. If this process was not extremely fast, the surround of BC receptive fields would lag the center when responding to moving stimuli. Conversely, reducing temporal redundancies requires a slow mechanism so that lasting activity can be subtracted from the cone output. The feedback system we present here fulfills these requirements. The ephaptic feedback mechanism is extremely fast and will be prominently involved in reducing spatial redundancies. The Panx1/ATP-mediated mechanism is especially suitable for reducing temporal redundancies. As the slow component of feedback can only contribute when relatively static stimuli are used, it will not compromise the fast spatial redundancy reduction via the ephaptic feedback mechanism.

### Inhibiting the Slow Component of Feedback

In a first order approximation it is expected that inhibiting the slow component of feedback will always reduce the amplitude of feedback. However, this is not necessarily the case. For example, enhancing the amount of pH buffer experimentally by application of ATP will keep the pH in the synaptic cleft low and so I_Ca_ remains inhibited. Because the pH in the cleft no longer depends on HC hyperpolarization, the slow component of feedback is lost and the total feedback response becomes smaller. In contrast, when the formation of the pH buffer is prevented by ARL67156, the pH in the synaptic cleft is again no longer dependent on HC hyperpolarization, but now the pH is high and I_Ca_ disinhibited. Because of this larger I_Ca_, the total feedback responses will increase, even though the slow component is lost. Indeed this is what was found experimentally (Figure 4B and C).

One would also expect that probenecid and ARL67156 would affect the feedback amplitude similarly, as both drugs decrease the pH buffer concentration in the synaptic cleft. However, this was not the case as probenecid decreased the total feedback response amplitude, whereas ARL67156 did not (Figure 4A, C, and J). The difference between probenecid and ARL67156 application is that probenecid also modulates the conductance of the Panx1 channels, whereas ARL67156 does not. This suggests that Panx1 channels also participate in the ephaptic component of negative feedback together with Cx hemichannels [7],[11],[12] and glutamate-gated channels [47]. Reducing the Panx1 conductance will affect both the fast and the slow feedback component, whereas application of ARL67156 only affects the slow component. Blocking the ephaptic part of feedback will lead to a shift of the activation potential of I_Ca_ to more positive potentials, whereas inhibiting the ATP release leads to alkalization and thus a shift to more negative potentials. The overall effect of inhibiting Panx1 channels will therefore depend on the relative strength of both effects. Our data show that application of probenecid indeed does not significantly shift I_Ca_, indicating that both Panx1-mediated feedback signals affect I_Ca_ equally but in opposite directions (Figure 4K).

### Feedforward and Feedback Keep Each Other Balanced

Why does probenecid have a greater effect on the HC membrane potential when the feedback is blocked with 28 mM HEPES? The cone-HC system is a closed loop. Glutamate release by cones sets the membrane potential of HCs (feedforward signal), whereas feedback from HC to cones determines the amount of glutamate released by the cones. In other words, the feedforward signal from cones to HCs is kept in its working range by the feedback signal from HCs to cones. They keep each other balanced. Therefore, it is expected that when feedback is blocked completely, I_Ca_ is no longer kept in its working range and cones stop releasing glutamate. Indeed this happens when feedback is blocked by carbenoxolone [6]. Carbenoxolone completely blocks feedback by closing both the Cx hemichannels and Panx1 channels. This causes a large shift of the activation potential of I_Ca_ to more positive potentials, which induces a strong reduction of the glutamate release by cones and hyperpolarization of HCs. Because cones no longer release glutamate, HC light responses are lost. The fact that a high concentration of HEPES does not strongly hyperpolarize HCs or completely block their light responses suggests that HEPES does not block feedback completely. This was confirmed by Fahrenfort et al. [16], who showed that 10 mM HEPES or more reduces feedback to about 40% of its maximum. The consequence is that in the presence of 28 mM HEPES, the remaining part of feedback can keep the cone-HC system in its working range and HC light responses remain (Figure 5C). When the ephaptic Panx1/ATP-mediated feedback component is inhibited as well by adding probenecid in the presence of HEPES, the cone-HC system can no longer remain in its working range. The cones stop releasing glutamate and HCs hyperpolarize strongly and lose their light responses. On the other hand, applying probenecid in the absence of HEPES has only a relatively minor effect on the HC membrane potential, as in this condition the Cx-hemichannel-mediated feedback pathway will not be affected and can keep the cone output in its working range and HC light responses remain present.

### The Role of Adenosine

Stella and co-workers [42]–[44] report that adenosine decreased I_Ca_ in cones via an A2 receptor interaction. In this article we showed that the slow component of feedback did not depend on this pathway. In fact, we found no effect at all on the feedback responses of cones when adenosine receptors were blocked. This disparity may have occurred as a result of the different experimental conditions used. We used relatively light-adapted goldfish or zebrafish retinas, whereas Stella and co-workers [42]–[44] worked with salamander retinas that were dark-adapted, a condition where adenosine signaling might be most prominent [48]. Furthermore, at present there is no direct evidence for the expression of adenosine receptors in the cone synaptic terminal, whereas expression is found in the inner retina [48]–[51]. It is possible that the effects seen by Stella and co-workers [42]–[44] are mediated by activation of A2 receptors in the inner retina affecting the outer retina via, for instance, interplexiform cells.

### How General Are These Inhibitory Mechanisms?

Are the feedback mechanisms we describe here also present in other vertebrates, or are they specific for fish? There is general agreement that in all vertebrates, ranging from salamander and fish to mice and primates, negative feedback from HCs modulates the I_Ca_ of cones [1]–[5]. In zebrafish, knocking out the hemichannel forming Cxs (Cx55.5) leads to a severe reduction of feedback from HCs to cones [7]. Interestingly, the rollback response remained intact [7]. Similarly, the HC rollback response is unaffected in mice when the HC-specific Cx (Cx57) is knocked out [52]. This led the authors to conclude that there was no Cx-hemichannel-mediated ephaptic feedback in mice. However, because the ephaptic feedback component is very fast and most likely does not contribute strongly to the rollback response in HCs, the conclusion that ephaptic feedback does not occur in mice might be premature. In the present study, we show that the rollback response in HCs, at least partly, depends on the slow Panx1/ATP-mediated component of feedback. Similar to zebrafish and goldfish, mice also express Panx1 channels at the HC dendrites [12]. It is therefore likely that in mice the slow component of feedback is also mediated by Panx1 and ATP.

It is tempting to speculate that both mechanisms are present in all vertebrates but in different ratios and that this ratio reflects a species' visual capability and needs. In some animals, the ephaptic component might be the largest, whereas in others the Panx1/ATP component might dominate. For example, as the spatiotemporal correlation function of natural scenes is inseparable [53], faster temporal signals received by the retina of species like mice with low visual acuity will be limited under normal conditions. In animals such as these, the slow Panx1/ATP component of feedback may dominate as their retina has less need to manage the faster temporal aspects of the scene. On the other hand, in animals strongly depending on vision, such as zebrafish and goldfish, the fast component might be dominating. Whether the ratios of the two feedback mechanisms indeed correlate with the visual performance of the various animals is an intriguing question that awaits further study.

Could the Panx1/ATP mechanism presented here also function outside the retina? In the central nervous system, Panx1 and NTPDase1 are both abundantly present and their localization is concentrated in synapses [54]–[56]. This suggests that the Panx1/ATP system described in this article might also occur in other brain regions. The hippocampus is one region where Panx1 expression levels are high, and recently it was suggested that Panx1 channels are involved in synaptic plasticity stabilization in that brain area, although the underlying mechanism has not been clarified [57]. As NMDA receptors are very sensitive to changes in pH [58], the proposed Panx1/ATP system may also be acting as a modulator of synaptic strength in the hippocampus and other brain regions.

Panx1 is also expressed in astrocytes and astrocytes are known to release ATP [27]. Processes of astrocytes and pre- and postsynaptic structures of neurons form a tripartite synapse. Astrocytes play an active role in such a complex; they modulate the signal flow between pre- and postsynaptic cells and in that way influence the neuronal network properties [59]. This modulation depends on the intracellular Ca^2+^ concentration in the astrocytes. Because Panx1 channels are gated by intracellular Ca^2+^, and because any voltage-gated channel, such as the presynaptic voltage-gated Ca channels, is pH sensitive due to pH-induced changes in surface charge, astrocytes might modulate the synaptic efficiency by changing the pH buffering in the synaptic cleft utilizing the Panx1/ATP system described in this article. Furthermore, because both Panx1 and NTPDase1 are also coexpressed in other organs like the kidneys and heart [21],[55],[60], similar extracellular pH modulation systems might also modulate cellular activity in nonneuronal tissues.

## Materials and Methods

### Experimental Animals and Isolated Retina Preparation

All animal experiments were carried out under the responsibility of the ethical committee of the Royal Netherlands Academy of Arts and Sciences acting in accordance with the European Communities Council Directive of November 24, 1986 (86/609/EEC). Goldfish, *Carassius auratus*, or zebrafish, *Danio rerio*, were euthanized and the eyes enucleated. Wild-type and Cx55.5 mutant zebrafish (C54X, hu1795, ZFIN ID: ZDB-ALT-110920-1), all in a TL background, were used.

### Electrodes and Recording Set-Up

#### Isolated retina preparation

Electrophysiological recordings of cones and HCs in the isolated retina were made following published methods [19].

Patch-pipettes (resistance 8–12 MΩ) were pulled from borosilicate glass capillaries (GC-150T-10, Harvard Apparatus Ltd, UK) with a Brown Flaming Puller (Model P-87; Sutter Instruments Company). Pipettes were connected to an Axopatch 200A patch clamp amplifier (Molecular Devices, Sunnyvale, CA; four-pole low-pass Bessel filter setting, 2 KHz) or a Dagan 3900 integrating patch clamp amplifier (Dagan Corporation, Minneapolis, MN). All data shown are corrected for the junction potential.

For the intracellular recordings of HCs, a WPI S7000A (WPI, Sarasota, FL) microelectrode amplifier system was used. Microelectrodes were pulled on a horizontal puller (Sutter P-80-PC; San Rafael, CA) using aluminosilicate glass (OD = 1.0 mm, ID = 0.5 mm; Clark, UK) and had impedances ranging from 70–200 MΩ when filled with 3 M KCl. Data were digitized and stored with a PC using a CED 1401 AD/DA converter at 4 KHz using Signal software [v. 3.07; Cambridge Electronic Design (CED), Cambridge, UK] to acquire data, generate voltage command outputs, and drive light stimuli. AxographX (v1.3.5, AxoGraph Scientific), Origin Pro (v8, Origin Lab Corporation), and Matlab (v2012b, MathWorks) were used for the analysis. All data are corrected for the liquid junction potential.

#### Dissociated HCs

After the dissociation procedure described below, cell suspension was placed on the recording chamber and cells were allowed to settle for 15 min. The gravity perfusion system allowed a changed of the bath solution for drug application. HCs were visualized using an upright microscope under normal white light. Pipette resistance was 8–12 MΩ. All data were acquired with a Dagan 3900 integrating patch clamp amplifier (Dagan Corporation, Minneapolis, MN). Igor.pro software (WaveMetrics Inc., Oregon) was used for the analysis. All data are corrected for the liquid junction potential.

Cells were held at −60 mV and currents were triggered by a change in the clamp potential from −100 mV to +100 mV, with 10 mV steps, for 200 ms. Data were acquired at a rate of 10 kHz and low-pass filtered at 2 kHz.

### Solutions

For the isolated retina preparation, control Ringer solution contained (in mM) 102.0 NaCl, 2.6 KCl, 1.0 MgCl_2_, 1.0 CaCl_2_, 28.0 NaHCO_3_, 5.0 glucose, and 0.1 picrotoxin and was continuously gassed with 2.5% CO_2_ and 97.5% O_2_ to yield a pH of 7.6. HEPES Ringer solution contained (in mM) 102.0 NaCl, 2.6 KCl, 1.0 MgCl_2_, 1.0 CaCl_2_, 28.0 HEPES, 5.0 glucose, and 0.1 picrotoxin, and the pH was set to 7.6 with NaOH. The pipette solution contained (in mM) 85 K-gluconate, 21 KCl, 1 MgCl_2_, 0.1 CaCl_2_, 1 EGTA, 10 HEPES, 10 ATP-K_2_, 1 GTP-Na_3_, 20 phosphocreatine-Na_2_, 50 units ml^−1^ creatine phosphokinase, adjusted with NaOH to pH 7.3, and resulting in a E_Cl_ of −50 mV when used in conjunction with the Ringer solution. All chemicals were supplied by Sigma-Aldrich (Zwijndrecht, the Netherlands), except for Papain (Worthington Biochemical Company, Lakewood, NJ) and ARL67156 and ZM241385 (Tocris Biosciences, Bristol, UK). For the electrophysiological experiments with dissociated HC, the following solutions were used. The control Ringer solution contained (in mM) 110 NaCl, 5 KCl, 10 CsCl, 2.5 CaCl_2_, 2 MgCl_2_, 10 HEPES, 10 Glucose, pH 7.8. Intracellular recording solution contained (in mM) 10 NaCl, 120 CsCl, 5 CaCl_2_ (free 100 µM), 5 EGTA, 10 HEPES, 2 ATP-Mg, pH 7.4. Concentrations of the drugs used are indicated in the legends.

### Luminescence Measurements

HCs of goldfish were isolated using an optimized version of the procedure originally described by Dowling et al. [23] and optimized by Ayoub and Lam [24]. Retinas were dissected as described above. The isolated retinas were placed in a solution of L-15 containing 5 mg/ml papain (Worthington, no. 3126). They were incubated at room temperature for 35 min while being shaken at a low frequency. Subsequently, the retinas were washed in DMEM+Glutamax (GIBCO, no. 61965) containing 10% fetal bovine serum (GIBCO, no. 10270) to inactivate the papain. Then the retinas were washed in L-15 and finally placed in a tube containing 3 ml L-15. The retinas were mechanically dissociated by trituration using a wide plastic Pasteur pipette first and a narrower fire polished glass pipette for later fractions. For each new fraction, the cells were allowed to settle for a minute before the lower heavier section was transferred to a new tube. The fractions containing an HC concentration of 85% or higher were used for luminescence measurements. ATP bioluminescence was measured using the ATP Bioluminescence Assay Kit CLS II purchased from Roche. Luciferase luminescence was measured with a Varioscan Flash (Thermo Scientific) using luminometry. After obtaining a baseline measure, 50 µM AMPA was added and subsequently 100 µM probenecid.

### Light Stimuli

A 20 µm white light spot (0 log) was focused via a 60× water immersion objective on the cone outer segment and a 4,500 µm “full field” white spot (−1.5 log) projected through the microscope condenser. The light stimulator consisted of two homemade LED stimulators based on a three-wavelength high-intensity LED (Atlas, Lamina Ceramics Inc., Westhampton, NJ). The peak wavelengths of the LEDs were 624, 525, and 465 nm, respectively, with bandwidths smaller than 25 nm. An optical feedback loop ensured linearity. The output of the LEDs was coupled to the microscope via light guides. White light consisted of an equal quantal output of the three LEDs. 0 log intensity was 8.5×10^15^ quanta m^−2^ s^−1^.

### Method for Measuring the Time Constants of Feedback

The full-field light onset responses of the negative feedback measurements were fitted with a single exponential:
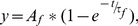
(1)and if possible with a double exponential function:
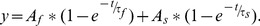
(2)To test which of the two models described the raw data best, an *F* test comparing the sum of squares of residuals of each fit was performed.

### Method for Measuring Pharmacological Effects on the Slow Component of Feedback

Often the slow component of feedback present in control conditions was reduced by our pharmacological manipulation to such a level that the onset of the feedback current could no longer be fitted accurately by a double exponential function. In order to quantify the effect of the various compounds used on the slow component of feedback, the average amplitudes were determined for 40 ms stretches of the feedback current centered at 160 ms and 460 ms after response onset, and the difference was calculated. This difference was taken as the amplitude of the slow component of feedback. The amplitude of the slow component in the pharmacological condition (B) was divided by the amplitude of the slow component in the control condition (A), yielding the reduction ratio.

### Method for Determining the Shift in I_Ca_ Half Activation Potential

The activation curve of I_Ca_ was derived by leak subtracting the IV relation using the linear part of the IV relation between −80 and −60 mV [16]. The half activation potential was then determined by fitting a Boltzmann relation (Eq. 3) through the leak subtracted IV relations:
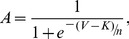
(3)where *V* is the membrane voltage, 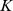 is the midpoint, and *m* is the slope factor. This was done for each cell in both control (C) and experimental (E) conditions and the shift in the half activation potential calculated as K(E) minus K(C).

### Immunohistochemistry

Immunohistochemical procedures were similar to published methods [7],[16]. The Panx1 antibody was raised against the zebrafish sequence and characterized by Prochnow et al. [11]. The primary antibody against NTPDase1 was raised in rabbit to the corresponding amino acid sequence 102–130 of human NTPDase1 (GIYLTDCMERAREVIPRSQHQETPVYLGA). It was obtained from CHUQ (www.ectonucleotidases-ab.com) and characterized by Ricattie et al. [61]. The ADA (AB176) and GluR2 antibodies were purchased from Chemicon International (Temecula, CA). The ADA rabbit polyclonal antibody was raised against calf spleen ADA. The GluR2 (MAB397) mouse monoclonal (clone 6C4) antibody was raised against a recombinant fusion protein TrpE–GluR2 (N-terminal portion, amino acids 175–430 of rat GluR2). Secondary antibodies, goat–anti-mouse Alexa 488, and goat–anti-rabbit Cy3 were purchased from the Jackson Immuno Research Lab (West Grove, PA).

For light microscopical (LM) purposes, 10-µm-thick sections were made and stored at −20°C. Sections were first preincubated in 2% Normal Goat Serum (NGS) for 30 min, then incubated with primary antibodies for 24–48 h, followed by 35 min of incubation with secondary antibodies at 37°C. The 0.25 µm optically sectioning was performed with Zeiss CLSM meta confocal microscope (Zeiss, Germany).

For electron microscopical (EM) purposes, 40-µm-thick sections were incubated with the primary antibody in PB for 48 h, then rinsed before being incubated with rabbit peroxidase antiperoxidase (PAP) for 2 h, rinsed, and then developed in a 2,2′-diaminobenzidine (DAB) solution containing 0,03% H_2_O_2_ for 4 min. Afterwards the gold substitute silver peroxidase method [62] was performed; sections were fixed in sodium cacodylate buffer (pH 7.4) containing 1% Osmium tetra oxide and 1.5% potassium ferricyanide. Sections were then dehydrated and embedded in Epoxy resin, ultrathin sections made and examined with a FEI Tecnai 12 electron microscope.

### Statistics

All data are presented as mean ± sem unless otherwise stated. Significance was tested using a two-tailed *t* test on paired or independent group means as appropriate.

## Supporting Information

Figure S1 Feedback responses in cones. (A) The feedback-induced modulation of I_Ca_ in cones leads to the activation of a Ca^2+^-dependent Cl^−^ current (I_Cl(Ca)_) [1],[19]. To prevent interference of I_Cl(Ca)_ with the measurements of the kinetics of the feedback responses, we clamped cones at E_Cl_ (*n* = 23) (A_ii_). To validate this method, cells were also clamped at E_Cl_+5 mV (*n* = 7), and at E_Cl_−5 mV (*n* = 13) (A_i_ and A_iii_) and I_Cl(Ca)_ was blocked with niflumic acid (*n* = 7), a relative specific blocker of I_Cl(Ca)_ (A_iv_). Mean feedback responses measured under these conditions all consisted of two processes, one dominating process with a time constant around 25 ms (τ_f_) and one with a large time constant (τ_s_) (B_i_ and B_ii_). The large time constant depended on E_Cl_. Feedback leads to the influx of Ca^2+^, activating I_Cl(Ca)_. The activation of this current did not interfere significantly with the amplitude of the feedback response (*p*>0.05), but it did interfere with the estimation of the time constant of the slow component. B_ii_ shows that as soon as the clamp potential deviates from E_Cl_, the time constant becomes either too large or too small. With the clamp potential and E_Cl_ equal, I_Cl(Ca)_ does not contribute any more. These experiments show that we could adequately remove the contribution of I_Cl(Ca)_ from our measurements.
(TIF)

Figure S2The fast feedback component is mediated via an ephaptic mechanism. What are the properties of the fast feedback component? Because feedback is driven by HCs, we have to compare the kinetics of the feedback response to those of the HC response. (A) An HC response to a full-field light flash (black, left) and a feedback response in a cone (red, middle) are superimposed (right). Note that the responses are scaled arbitrarily to each other. The light onset responses of both HCs and feedback fully overlap, suggesting no strong filtering by the feedback synapse and the absence of a synaptic delay in the feedback pathway. Interestingly, the feedback response does not show the slow rollback characteristics (arrow) present in the HC response. This is because the slow component of feedback adds to the total feedback response, while it will be inhibitory in the HC response. To obtain a better estimate of the temporal properties of the feedback synapse, we derived the frequency transfer function of the feedback synapse. To measure the possible delay between the HC response and the feedback response, a linear systems analysis approach was followed. A mixed sinusoid stimulus was used to stimulate the retina (see the methods below). (B) This stimulus contains 17 sinusoids with frequencies ranging from 0.5 to 31.75 Hz. By combining all sinusoids, the mean intensity as well as the mean temporal contrast remains equal. The stimulus was presented about 11 times and the mean responses were determined. The transfer function between the mixed sine stimulus (B) and either the HC responses (*n* = 7) (C, black line) or the feedback responses (n = 6) (D, red line) were determined. Convolving the transfer functions with the stimulus predicted 97±1% of the light-dependent structure [65] for both HC (*n* = 7) and feedback (*n* = 6) responses (C and D, green lines). The green traces show that the linear prediction for these responses almost completely overlap with the original HC, and feedback responses (black trace) indicating that both systems were well described by their transfer functions under these stimulus conditions. The HC and feedback responses were used to derive the gain/frequency and phase/frequency relations (E and F). The gain/frequency curves overlap completely in the high frequency range, indicating that feedback to cones occurs without additional temporal filtering (−3 dB cutoff frequencies; feedback, 9.3±1.6 Hz; HC, 7.6±1.2 Hz; *p*>0.15). At low frequencies, the two frequency/response curves are significantly different, showing the involvement of the slow feedback component. Phase differences can be interpreted as delays. The phase/frequency curves overlap completely, indicating that there is no synaptic delay between the HC response and the feedback response. This was further quantified in (G). We determined that the delay between the HC and the feedback responses for frequencies above 9 Hz was 0.073±0.842 ms (Figure 2G, gray area), which does not differ significantly from zero (*p*>0.5). Finally, we compared the mean impulse functions of the HC signal (black) and the feedback signal (red) (H). The gray area around the curves indicates the sem. As expected the impulse functions overlapped almost completely except at the later time points. The HC impulse function and the feedback impulse function did not differ significantly in onset time or time to peak. These experiments indicate that HC to cone feedback is very fast and has no delay. These features are consistent with an ephaptic feedback mechanism mediated by Cx hemichannels [6],[7],[16], making this synapse one of the fastest inhibitory processes in the nervous system. *Methods for the time delay estimation*: The stimulus used was the sum of sine waves with a frequency of 0.5, 0.75, 1.25, 2.75, 3.25, 4.25, 4.75, 5.75, 7.25, 9.25, 10.75, 14.75, 17.75, 20.75, 24.25, 27.25, and 31.75 Hz with equal amplitude (Michelson contrast 100%) and randomized phase. The resulting stimulus had a mean intensity of −1.5 log and temporal contrast of 0.33 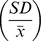. Frequency values were chosen such that higher frequencies were not harmonics of lower frequencies, and spectral leakage between measured frequencies and contamination by equipment noise (typically whole number frequencies) were minimized. The 4 s stimulus (S) and responses (R) data were first mean subtracted and Fourier transformed. The length of the Fourier transform was set such that each frequency used in generating the mixed sine stimulus was resolved. The transfer function [F_sr_(f)] (Eq. S4) was calculated as the quotient of the cross-power spectral density of S(f) and R(f) and the power spectral density of S(f):
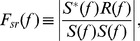
(S1)where * is the complex conjugate and | | denotes averaging over multiple stimulus presentation repeats (mean number of stimulus repeats: feedback, 12±1.2; HC, 11±1.8). HC responses and feedback responses to the mixed sine stimulus were well described by their transfer functions. The average correlations between a cell's mean response and its individual responses were 0.93±0.01 (*n* = 6) for feedback and 0.97±0.02 (*n* = 7) for HCs. The average correlations between a cell's individual responses and the response predicted by convolving the stimulus with its transfer function were 0.91±0.01 for feedback and 0.94±0.01 for HCs. These linear filters therefore described 96.9±0.5% and 96.6±0.5% of the light-dependent structure of feedback and HC responses, respectively [65]. To assess if there was a synaptic delay between the HC responses and feedback responses, we used their transfer function phase responses to calculate and compare their delay and group delay characteristics. The delay gives the time delay of each sinusoidal component of the stimulus in either the HC responses or feedback responses, whereas the group delay gives the average time delay experienced over a range of frequencies. For these calculations, we first converted the temporal frequencies (Hz) of interest to angular frequencies (rad/s), for computational simplicity:
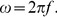
(S2)The delay for each frequency was calculated as:
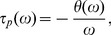
(S3)where *τ_p_*(*ω*) is the delay in seconds at frequency *ω*; *θ*(*ω*) is the phase response in radians of the stimulus/response transfer function at frequency *ω*; *ω* is the angular frequency in radian per second. The group delay (in seconds) is the negative first derivative of the transfer function phase response. We generated the average derivative for both the HC and feedback datasets by a linear regression through the origin fit through all their respective transfer function phase responses [66]. We limited the frequency range in these regressions to include only the higher frequencies (58.11 and 199.49 rad/s corresponding to the temporal frequencies: 9.25 to 31.75 Hz) where the phase response for both feedback and HCs was very linear. Using these criteria, the regression analyses for feedback contained 48 data points, and for HCs it was 56 (i.e., 8 data points each from the six feedback and seven HCs responses). The resulting regression coefficients correspond to the first derivative (feedback, −0.072332±0.000544, r^2^ = 0.997; HCs, −0.072405±0.000615, r^2^ = 0.996), equating to an average group delay over the frequency range assessed for feedback of 72.3±0.54 ms and for HCs of 72.4±0.62 ms. To calculate the mean group delay difference between feedback and HCs responses and assess if this difference was statistically significant, we compared the equality of the two regression coefficients in the following way. The difference in the regression slopes, which equates to a difference in group delay, is:
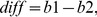
(S4)where *b*1 and *b*2 refer to the regression coefficients. The standard error of the difference between the regression coefficients is:
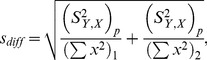
(S5)where 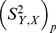
is the pooled residual mean square:
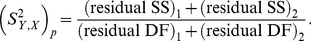
(S6)The test statistic used is:
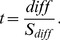
(S7)The degrees of freedom (*v*) were calculated as follows:
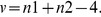
(S8)
(TIF)

Figure S3 To rule out possible other effects of probenecid than inhibiting Panx1, we investigated the presence of organic anion transporters (OATs) in the retina. The only other proteins known to be inhibited by probenecid are OATs [67]. OAT1 is expressed in the brain [68], but retinal expression in fish has not been assessed. To test whether this transporter is also expressed in the retina, we performed an in situ hybridization (ISH) experiment. Here we show with ISH of a probe directed to OAT1 that the OAT1 was expressed in various places in the brain. (A) Horizontal slice through the zebrafish brain at the level of the telencephalon and mesencephalon. The blue reaction product is widely present. Note for instance the restricted presence of reaction product in a layer in the tectum opticum. Scale bar, 500 µm. Images were acquired with a 20× objective and an Evolution MP Color camera (Media Cybernetics, Rockville, USA) connected to an Axioskop light microscope (Zeiss, Göttingen, Germany) and stitched together with Image Pro 6.3. The insert in panel (A) shows that the label is exclusively present in the cellular compartments. Scale bar for the insert is 25 µm. No reaction product was found in the inner and outer nuclear and ganglion cell layers of the zebrafish retina (B). Scale bar, 50 µm. Thus, probenecid can be considered a specific Panx1 inhibitor in the retina, as its actions are not mediated via an OAT1 pathway. (C) The mean whole-cell IV relations of four dissociated HCs in control conditions (black) and when the Panx1 current was blocked with 20 µM BB FCF (red). During the whole experiment, potassium currents were inhibited by Cs. The green trace is the IV relation of the BB FCF blocked current. This current has the similar characteristics as the Panx1 current described by Prochnow et al. [11]. Both (B) and Figure S3C demonstrate current flows via Panx1 channels within the physiological membrane potential range. *Methods ISH*: Zebrafish brain and eyes were isolated and fixed in 4% formalin solution for 2 h, treated with a sucrose solution to prevent ice crystal formation in storage, and fixed for another 24 h in 25% paraformaldehyde (PFA). The tissue was embedded in Tissue-Tek and frozen. Cryostat sections of 10 µm were collected with a Leica CM 3050 S cryostat (Leica Biosystems, USA) and stored at −80°C. For ISH, sections were stored at −80°C. Hybridization was performed using OAT1-specific, 5′-fluorescein-labeled 25 mer antisense oligonucleotides containing locked nucleic acid (LNA) (base uppercase) and 2′-O-methyl (2OME)-RNA moieties (base lowercase); OAT1-1 (5′-Fam/TagTagGcaAagCtgGtgCtgGagA-3′). Probes were synthesized by Exiqon (Odense, Denmark). Hybridization signals were detected by incubating the sections in blocking buffer containing anti-fluorescein-alkaline phosphatase (AP) Fab fragments (1∶1,000; Roche, Lewes, UK) for 1 h at room temperature. AP signal was detected by using a substrate kit (Vector Blue AP Substrate Kit III; Vector, Burlingame, CA).
(TIF)

Figure S4 Individual cone feedback responses in various pharmacological conditions. (A) Application of 500 µM probenecid blocks the slow component of feedback and decreases the feedback amplitude (black, control; red, probenecid; green, wash). (B) Application of 100 µM ATP blocks the slow component of feedback and decreases the feedback amplitude (black, control; red, ATP; green, wash). (C) Application of 50 µM ARL67158 blocks the slow component of feedback and increases the feedback amplitude (black, control; red, ARL67158; green, wash). (D) Shifting the pH of the extracellular medium to pH 7.2 blocks the slow component of feedback and reduces the feedback amplitude (black, pH 7.6; red, pH 7.2; green, wash).
(TIF)

## Abbreviations

ADA: adenosine deaminase
ADP: adenosine diphosphate
AMP: adenosine monophosphate
AMPA: α-Amino-3-hydroxy-5-methyl-4-isoxazolepropionic acid
ARL67156: 6-N,N-Diethyl-D-β,γ-dibromomethylene
ATP: adenosine triphosphate
BB FCF: brilliant blue FCF
BFA1: bafilomycin A1
Cx: connexin
E_Cl_: chloride equilibrium potential
GABA: gamma-aminobutyric acid
GluR: glutamate receptor
HC: horizontal cell
HEPES: 4-(2-Hydroxyethyl)piperazine-1-ethanesulfonic acid
I_Ca_: Ca^2+^ current
I_Cl(ca)_: Ca-dependent Cl current
IR: immunoreactive
IV relation: current–voltage relation
NMDA: N-methyl-D-aspartate
NTDPase: Ecto-nucleoside triphosphate diphosphohydrolase
OAT: organic anion transporter
OPL: outer plexiform layer
Pnx1: Pannexin 1
V-ATPase: vacuolar H^+^-ATPase
WT: wild-type
ZM241385: 4-(2-[7-Amino-2-(2-furyl)[1,2,4]triazolo[2,3-*a*][1,3,5]triazin-5-ylamino]ethyl)phenol
