# Supplementary figures and images for: Extracellular ATP Hydrolysis Inhibits Synaptic Transmission by Increasing pH Buffering in the Synaptic Cleft
Source: PLoS Biol. 2014 May 20;12(5):e1001864. doi: 10.1371/journal.pbio.1001864 (PMC4028192; doi:10.1371/journal.pbio.1001864)

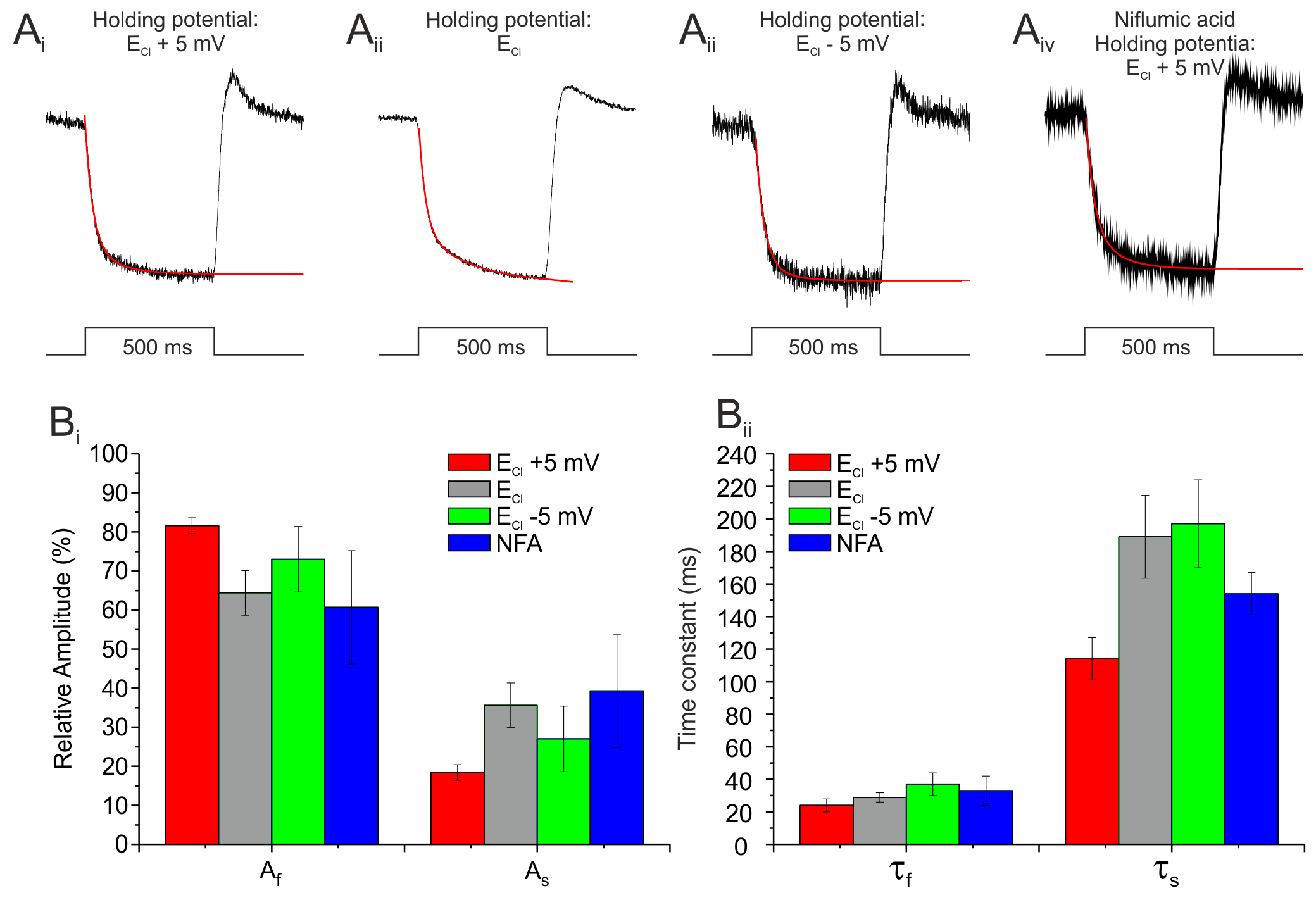

Supplement: Figure S1 — Feedback responses in cones. (A) The feedback-induced modulation of ICa in cones leads to the activation of a Ca2+-dependent Cl− current (ICl(Ca)) [1],[19]. To prevent interference of ICl(Ca) with the measurements of the kinetics of the feedback responses, we clamped cones at ECl (n = 23) (Aii). To validate this method, cells were also clamped at ECl+5 mV (n = 7), and at ECl−5 mV (n = 13) (Ai and Aiii) and ICl(Ca) was blocked with niflumic acid (n = 7), a relative specific blocker of ICl(Ca) (Aiv). Mean feedback responses measured under these conditions all consisted of two processes, one dominating process with a time constant around 25 ms (τf) and one with a large time constant (τs) (Bi and Bii). The large time constant depended on ECl. Feedback leads to the influx of Ca2+, activating ICl(Ca). The activation of this current did not interfere significantly with the amplitude of the feedback response (p>0.05), but it did interfere with the estimation of the time constant of the slow component. Bii shows that as soon as the clamp potential deviates from ECl, the time constant becomes either too large or too small. With the clamp potential and ECl equal, ICl(Ca) does not contribute any more. These experiments show that we could adequately remove the contribution of ICl(Ca) from our measurements. (TIF) [file pbio.1001864.s001.tif]

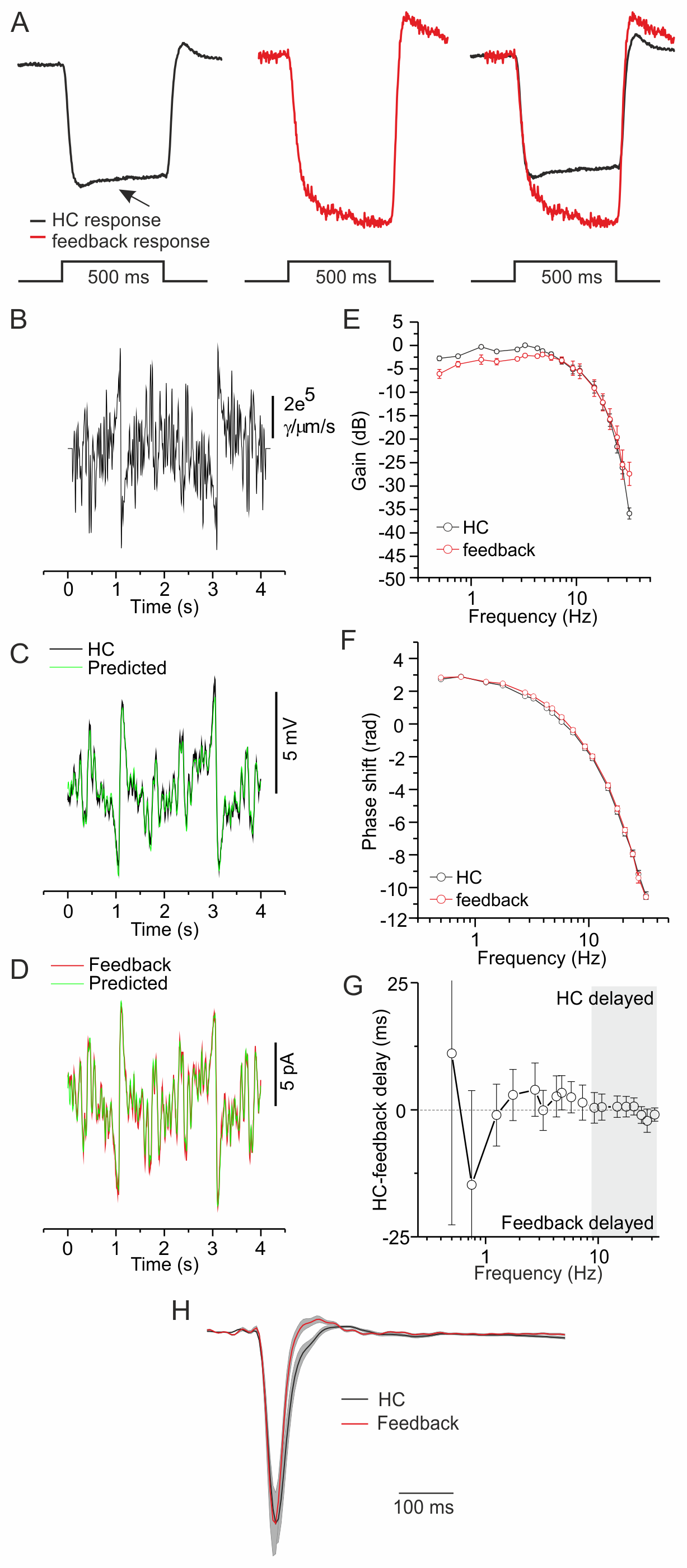

Supplement: Figure S2 — The fast feedback component is mediated via an ephaptic mechanism. What are the properties of the fast feedback component? Because feedback is driven by HCs, we have to compare the kinetics of the feedback response to those of the HC response. (A) An HC response to a full-field light flash (black, left) and a feedback response in a cone (red, middle) are superimposed (right). Note that the responses are scaled arbitrarily to each other. The light onset responses of both HCs and feedback fully overlap, suggesting no strong filtering by the feedback synapse and the absence of a synaptic delay in the feedback pathway. Interestingly, the feedback response does not show the slow rollback characteristics (arrow) present in the HC response. This is because the slow component of feedback adds to the total feedback response, while it will be inhibitory in the HC response. To obtain a better estimate of the temporal properties of the feedback synapse, we derived the frequency transfer function of the feedback synapse. To measure the possible delay between the HC response and the feedback response, a linear systems analysis approach was followed. A mixed sinusoid stimulus was used to stimulate the retina (see the methods below). (B) This stimulus contains 17 sinusoids with frequencies ranging from 0.5 to 31.75 Hz. By combining all sinusoids, the mean intensity as well as the mean temporal contrast remains equal. The stimulus was presented about 11 times and the mean responses were determined. The transfer function between the mixed sine stimulus (B) and either the HC responses (n = 7) (C, black line) or the feedback responses (n = 6) (D, red line) were determined. Convolving the transfer functions with the stimulus predicted 97±1% of the light-dependent structure [65] for both HC (n = 7) and feedback (n = 6) responses (C and D, green lines). The green traces show that the linear prediction for these responses almost completely overlap with the original HC, and feedback respons [file pbio.1001864.s002.tif]

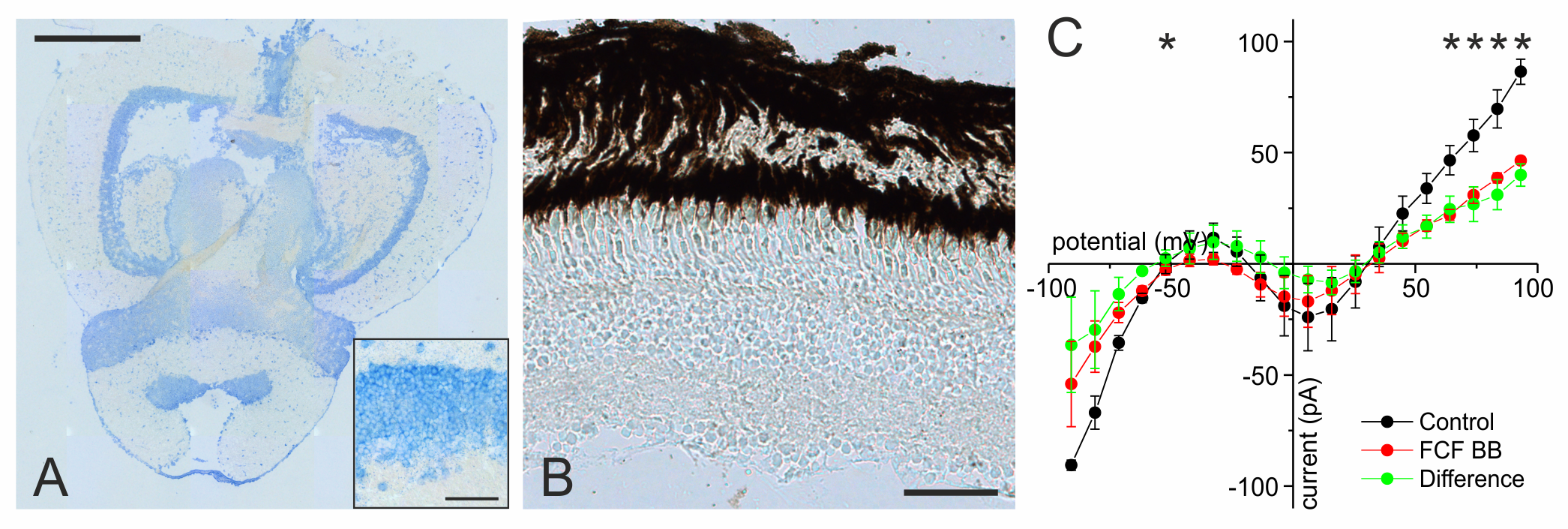

Supplement: Figure S3 — To rule out possible other effects of probenecid than inhibiting Panx1, we investigated the presence of organic anion transporters (OATs) in the retina. The only other proteins known to be inhibited by probenecid are OATs [67]. OAT1 is expressed in the brain [68], but retinal expression in fish has not been assessed. To test whether this transporter is also expressed in the retina, we performed an in situ hybridization (ISH) experiment. Here we show with ISH of a probe directed to OAT1 that the OAT1 was expressed in various places in the brain. (A) Horizontal slice through the zebrafish brain at the level of the telencephalon and mesencephalon. The blue reaction product is widely present. Note for instance the restricted presence of reaction product in a layer in the tectum opticum. Scale bar, 500 µm. Images were acquired with a 20× objective and an Evolution MP Color camera (Media Cybernetics, Rockville, USA) connected to an Axioskop light microscope (Zeiss, Göttingen, Germany) and stitched together with Image Pro 6.3. The insert in panel (A) shows that the label is exclusively present in the cellular compartments. Scale bar for the insert is 25 µm. No reaction product was found in the inner and outer nuclear and ganglion cell layers of the zebrafish retina (B). Scale bar, 50 µm. Thus, probenecid can be considered a specific Panx1 inhibitor in the retina, as its actions are not mediated via an OAT1 pathway. (C) The mean whole-cell IV relations of four dissociated HCs in control conditions (black) and when the Panx1 current was blocked with 20 µM BB FCF (red). During the whole experiment, potassium currents were inhibited by Cs. The green trace is the IV relation of the BB FCF blocked current. This current has the similar characteristics as the Panx1 current described by Prochnow et al. [11]. Both (B) and Figure S3C demonstrate current flows via Panx1 channels within the physiological membrane potential range. Methods ISH: Zebrafish brain and eyes were isolated and [file pbio.1001864.s003.tif]

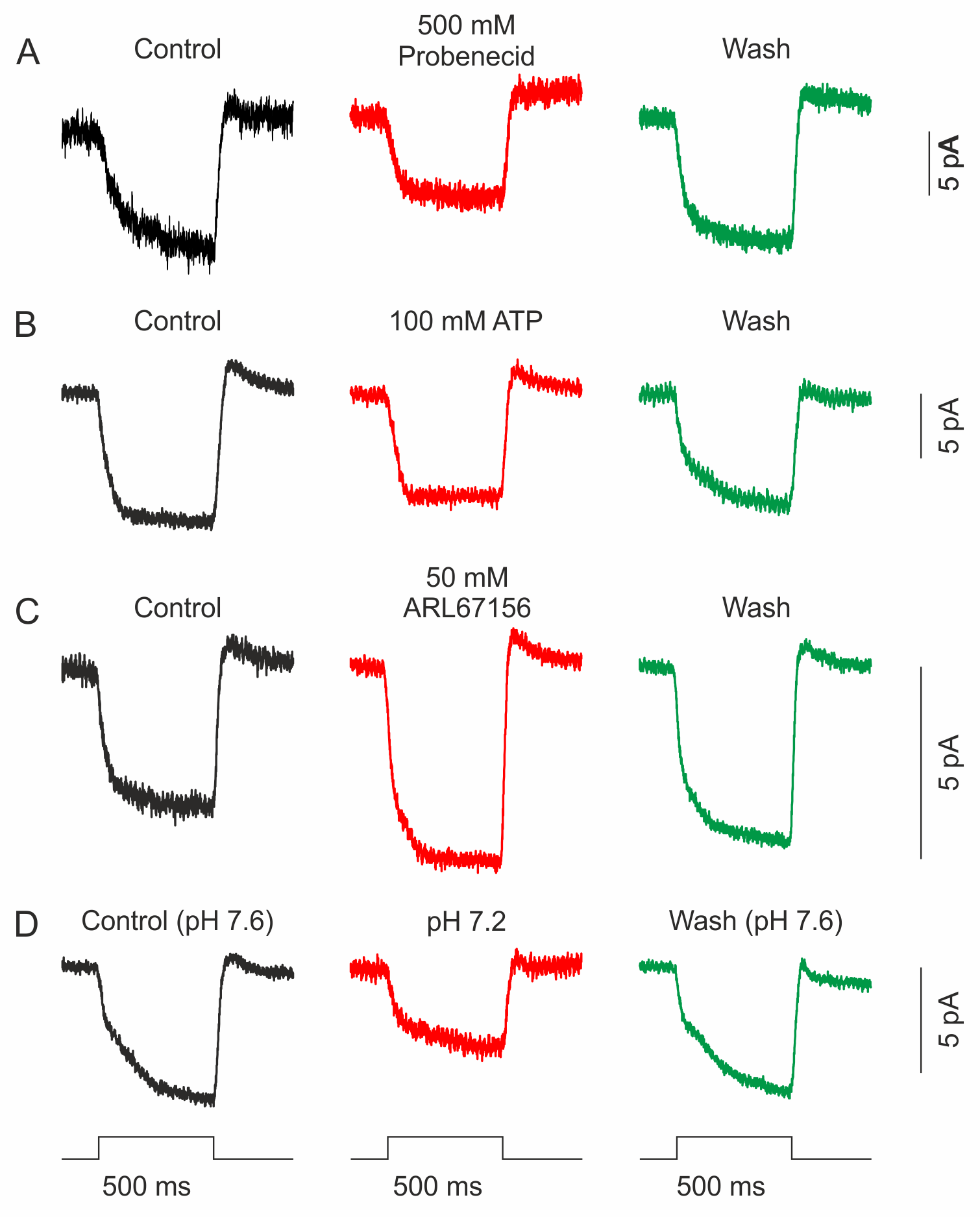

Supplement: Figure S4 — Individual cone feedback responses in various pharmacological conditions. (A) Application of 500 µM probenecid blocks the slow component of feedback and decreases the feedback amplitude (black, control; red, probenecid; green, wash). (B) Application of 100 µM ATP blocks the slow component of feedback and decreases the feedback amplitude (black, control; red, ATP; green, wash). (C) Application of 50 µM ARL67158 blocks the slow component of feedback and increases the feedback amplitude (black, control; red, ARL67158; green, wash). (D) Shifting the pH of the extracellular medium to pH 7.2 blocks the slow component of feedback and reduces the feedback amplitude (black, pH 7.6; red, pH 7.2; green, wash). (TIF) [file pbio.1001864.s004.tif]
